# The Complete Sequence of the First *Spodoptera frugiperda Betabaculovirus* Genome: A Natural Multiple Recombinant Virus

**DOI:** 10.3390/v7010394

**Published:** 2015-01-20

**Authors:** Paola E. Cuartas, Gloria P. Barrera, Mariano N. Belaich, Emiliano Barreto, Pablo D. Ghiringhelli, Laura F. Villamizar

**Affiliations:** 1 Centro de investigación Tibaitatá, Corporación Colombiana de Investigación Agropecuaria CORPOICA, Km 14 Vía Mosquera 250047, Cundinamarca, Colombia; E-Mails: pcuartas@corpoica.org.co (P.E.C.); lvillamizar@corpoica.org.co (L.F.V.); 2 Laboratorio de Ingeniería Genética y Biología Celular y Molecular—Área Virosis de Insectos (LIGBCM—AVI), Dto. de Ciencia y Tecnología, Universidad Nacional de Quilmes, Roque Saenz Peña 352, Bernal, Provincia de Buenos Aires, 1876, Argentina; E-Mails: mbelaich@unq.edu.ar (M.N.B.); pdg@unq.edu.ar (P.D.G.); 3 Centro de Bioinformática, Instituto de Biotecnología, Universidad Nacional de Colombia. Avenida Carrera 30 # 45, Bogotá 11001000, Cundinamarca, Colombia; E-Mail: ebarretoh@unal.edu.co

**Keywords:** baculovirus, granulovirus, *Spodoptera frugiperda*, genome, recombination

## Abstract

*Spodoptera frugiperda* (Lepidoptera: Noctuidae) is a major pest in maize crops in Colombia, and affects several regions in America. A granulovirus isolated from *S. frugiperda* (SfGV VG008) has potential as an enhancer of insecticidal activity of previously described nucleopolyhedrovirus from the same insect species (SfMNPV)*.* The SfGV VG008 genome was sequenced and analyzed showing circular double stranded DNA of 140,913 bp encoding 146 putative ORFs that include 37 *Baculoviridae* core genes, 88 shared with betabaculoviruses, two shared only with betabaculoviruses from Noctuide insects, two shared with alphabaculoviruses, three copies of own genes (paralogs) and the other 14 corresponding to unique genes without representation in the other baculovirus species. Particularly, the genome encodes for important virulence factors such as 4 chitinases and 2 enhancins. The sequence analysis revealed the existence of eight *homologous regions* (*hrs*) and also suggests processes of gene acquisition by horizontal transfer including the SfGV VG008 ORFs 046/047 (paralogs), 059, 089 and 099. The bioinformatics evidence indicates that the genome donors of mentioned genes could be alpha- and/or betabaculovirus species. The previous reported ability of SfGV VG008 to naturally co-infect the same host with other virus show a possible mechanism to capture genes and thus improve its fitness.

## 1. Introduction

The fall armyworm (FAW), *Spodoptera frugiperda* (J.E. Smith, 1797) (Lepidoptera: Noctuidae) is a polyphagous insect of wide geographical distribution, considered the most important pest in maize (*Zea mays* L.) in the Americas [1]. The larvae consume the plant whorl affecting its growth, and complete defoliation could arise when epizooties occur. Control of *S. frugiperda* in maize crops includes the integration of cultural, physical, biological and chemical methods [2]. However, the control with broad spectrum and highly toxic synthetic chemical insecticides (categories I and II) is the main method for reducing the effects of the pest [3]. To reduce the damage and maintain pest levels below the economic threshold, some strategies, such as biological control, have been developed through the use of entomopathogenic viruses, mainly from *Baculoviridae* family or baculovirus, principally of the genus *Alphabaculoviridae* [4,5,6]*.*

*Baculoviridae* is a family comprising rod-shaped viruses that infects members of the Phylum Arthropoda. This family includes four genera: *Alphabaculovirus* [lepidopteran-specific nucleopolyhedroviruses (NPVs)], *Betabaculovirus* [lepidopteran-specific Granuloviruses (GVs)], *Gammabaculovirus* (hymenopteran-specific NPVs) and *Deltabaculovirus* (dipteran specific NPVs) [7,8,9]. Their genomes vary in size from approximately 81.7 to 178.7 kbp according to available information, are circular covalently closed double stranded DNA (cccdsDNA) and encode ~90 to ~180 open reading frames (ORFs) [10]. The viral cycle presents a biphasic infection process generating progeny with two different phenotypes: budded viruses (BVs), which are produced at the initial stage of the multiplication cycle and are responsible for systemic infection inside the insect host, and occlusion-derived viruses (ODVs), which are produced in the last stage of the cycle within the infected cell and are required for the primary infection that takes place in the midgut epithelium cells of the insect host [9,11,12]. Mature ODVs are finally occluded in a protein matrix (mainly conformed by polyhedrin in alpha-, gamma- and deltabaculoviruses; or mainly conformed by granulin in betabaculoviruses) to form occlusion bodies (OBs), which protect them from the environment [13,14].

The use of mixtures containing granulovirus and nucleopolyhedrovirus has been studied in order to observe a synergic effect in the co-infections and in some cases granuloviruses are able to enhance the infectivity and virulence of NPVs [15,16]. This could be used as strategy for development of a biopesticide to control the FAW in maize crop in America. For this purpose, two baculoviruses (GV and NPV) were isolated from *S. frugiperda* larva collected in a pasture crop in Colombia (Córdoba) causing a natural co-infection [17]. The Colombian granulovirus (SfGV VG008) was characterized in morphologically, biologically and molecularly terms showing its potential use as enhancer of insecticidal activity of a SfMNPV [4,18]. SfGV VG008 was compared with a Brazilian SfGV isolate without finding differences in the insecticidal activity; however, the genomic comparison with restriction profiles showed differences in number and size fragments. To date, no information about the SfGV complete genome exists and there are only 16 full-length *Betabaculovirus* genomes with respect to 57 reports for *Alphabaculovirus* species [19]. Betabaculovirus genomes range from between 99,000–180,000 bp encoding between 119 and 183 putative proteins. Particularly, 14 articles reporting GV genome analyses were published since 1999 increasing knowledge of this kind of baculoviruses [20,21,22,23,24,25,26,27,28,29,30,31,32].

To add information that collaborates the understanding of the value of co-infections on insecticidal activity against *S. frugiperda*, the genome sequence and its bioinformatics characterization of SfGV VG008 are presented. In view of that, the genome analysis of SfGV VG008 revealed the presence of several ORFs that encode virulence factors such as enhancins and chitinases. Additionally, this betabaculovirus shows evidence of multiple points of recombination with both alpha- and betabaculoviruses, suggesting one of the main processes involved in baculovirus evolution.

## 2. Materials and Methods

### 2.1. Insect’s Source, Rearing and Virus Production

#### 2.1.1. Larvae of *S. frugiperda*

Larvae of *S. frugiperda* were obtained from a laboratory colony established in the Biological Control Laboratory of the Colombian Corporation of Agricultural Research (CORPOICA) using larvae collected from maize fields in Villavicencio, Colombia. This insect colony was periodically refreshed with insects collected in field and maintained at 25 °C, 60% RH (relative humidity) and 12:12 h (light:dark) photoperiod on a wheat germ-based semisynthetic diet [33].

#### 2.1.2. Occlusion Body Purification

Granulovirus SfGV VG008 was isolated from one *S. frugiperda* larva collected in a pasture crop in Colombia. For virus isolation, neonate larvae of *S. frugiperda* were inoculated using the droplet feeding method [34]. For this purpose, starved larvae of *S. frugiperda* were orally inoculated with an occlusion body (OBs) suspension (10^6^ OBs/mL), individually reared at 25 °C and 60% RH, under a natural photoperiod of 12:12 h (light:dark) until death. OBs were extracted from dead diseased larvae by homogenizing cadavers in 0.1% SDS solution (w/v) and purified by filtration and centrifugation on a 30%, 50% and 70% sucrose gradient [35]. To quantify viral suspensions absorbance measurements at 280 nm were carried out [18].

### 2.2. SfGV VG008 Genome Sequencing

Purified granules were dissolved by alkaline lysis and DNA was extracted according to Caballero *et al.* [36]. SfGV VG008 was sequenced using the 454 Genome Sequencer (GS) FLX™ Standard (Roche) at the Centro Nacional de Secuenciación Genómica (CNSG; Universidad de Antioquia, Medellín, Colombia), with consensus accuracy of Q20. *De novo* assembly was generated on NewBler assembler (GS FLX Data Analysis Software, Branford, Connecticut, USA) obtaining 1 unique contig of 140,917 bp. To verify some loci (the junction of the contig’s ends and other two regions with low quality data), PCR amplification, molecular cloning and subsequent sequencing by the standard dideoxy method of Sanger were performed. Thus, primers used were: V008_gap_122212_Fw (5'-CAT GGTTGTGCCAAAGTCAG-3') and V008_gap_122916_Rv (5'-GTCCATAGAGGACGGGTTGA-3'); V008_gap_109661_Fw (5'-TTGTGTTTCGCAATCTTCACCTTG-3') and V008_gap_109967_Rv (5'-GAGTATCACGAGTGCCGAGATG-3'); V008_gap_138941_Fw (5'-TGCGTGTTGGACACCGT TGT-3') and V008_gap_139260_Rv (5'-TGACCATAGTGACCAGTCTTGT-3'). This strategy allows confirming and correcting previous sequences, including the addition of two nucleotides and the elimination of other six. All the experiments associated with cloning were done using pGEM-T-Easy vector (Promega, Madison, Wisconsin, USA) and standard protocols.

ORFs were identified using ARTEMIS [37] and employing Fickett’s method [38]. ATG initiated ORFs of at least 150 nt (50 amino acids) with minimal overlap were selected for further analysis. All SfGV VG008 putative genes were searched for typical promoter motifs using *ad hoc* software (P.D. Ghiringhelli, 2012, unpublished) and previous data for the *Baculoviridae* family [22]. Initially the early CA(G/T)T and late (A/G/T)TAAG initiator (INR) sequences were searched in a sequence space comprising 120 residues upstream the ATG codon. After, a typical TATA box [TATA(A/T)A(A/T)] was searched in genes having an INR motif (25 to 35 bp upstream the INR) and in the upstream sequence of genes in which any INR was detected.

### 2.3. Phylogenetic Inference for SfGV VG008

#### 2.3.1. ORF Identification

In general, ORFeome and proteome similarity searches were done using BlastN, BlastP, tBlastN, tBlastX and PSI-Blast [39] initially against other betabaculovirus genomes and then against the other baculovirus species. Identities and similarities among homologous genes were obtained by doing global alignments with ClustalX [40,41] using default parameters.

#### 2.3.2. Phylogeny

Phylogeny was inferred using 37 core proteins [42] from 73 baculovirus genomes [43], plus data from SfGV VG008. Each core protein set was independently aligned using ClustalX program [40,41] with the following parameters: Pairwise alignment (Gap Open Penalty = 10, Gap Extension Penalty = 0.1, protein weight matrix: Gonnet 250); Multiple alignment (Gap Open Penalty=10, Gap Extension Penalty = 0.05, protein weight matrix: Gonnet series). Then, a concatemer was generated by addition of the complete individual alignments and phylogeny was inferred using MEGA program [44,45] with the following parameters: Method = Neighbor-Joining; Bootstrap with 1000 replicates; gap/Missing data = pairwise deletion; Model = Amino (Dayhoff Matrix); patterns among sites = Same (Homogeneous); rates among sites = Different (Gamma Distributed); gamma parameter = 0.8764. On the other hand, betabaculovirus core proteins were aligned among them using an all-against-all pairwise strategy, and identity and similarity percentages were obtained. 

Baculovirus genomes used are listed (Table 1).

**Table 1:** Baculovirus genomes used in the phylogenetic analysis.

Baculovirus	Acc. Number	Abbreviation
*Antheraea pernyi* MNPV Isolate L2	EF207986	AnpeMNPV
*Antheraea pernyi* NPV Isolate Z	NC_008035	AnpeNPV
*Anticarsia gemmatalis* MNPV	NC_008520	AgMNPV
*Autographa californica* MNPV Clone C6	NC_001623	AcMNPV
*Bombyx mandarina* NPV S1	NC_012672	BomaNPV S1
*Bombyx mandarina* NPV S2	JQ071499	BomaNPV S2
*Bombyx mori* NPV Isolate T3	NC_001962	BmNPV
*Choristoneura fumiferana* MNPV	NC_004778	CfMNPV
*Choristoneura fumiferana* Defective MNPV	NC_005137	CfDEFMNPV
*Choristoneura murinana* NPV Strain Darmstadt	NC_023177	ChmuNPV
*Choristoneura occidentalis* NPV Isolate BC1	NC_021925	ChocNPV
*Choristoneura rosaceana* NPV Isolate NB1	NC_021924	ChroNPV
*Epiphyas postvittana* NPV	NC_003083	EppoNPV
*Hyphantria cunea* NPV	NC_007767	HycuNPV
*Maruca vitrata *NPV	NC_008725	MaviNPV
*Orgyia pseudotsugata* MNPV	NC_001875	OpMNPV
*Philosamia cynthia ricini* NPV	JX404026	PhcyNPV
*Plutella xylostella* MNPV Isolate CL3	NC_008349	PlxyMNPV
*Rachiplusia ou* MNPV	NC_004323	RoMNPV
*Thysanoplusia orichalcea* NPV P2	NC_019945	ThorNPV P2
*Adoxophyes honmai* NPV	NC_004690	AdhoNPV
*Adoxophyes orana* NPV	NC_011423	AdorNPV
*Agrotis ipsilon* MNPV	NC_011345	AgipMNPV
*Agrotis segetum* NPV	NC_007921	AgseNPV
*Apocheima cinerarium* NPV	NC_018504	ApciNPV
*Buzura suppressaria* NPV Isolate Hubei	NC_023442	BusuNPV
*Chrysodeixis chalcites* NPV	NC_007151	ChchNPV
*Clanis bilineata* NPV Isolate DZ1	NC_008293	ClbiNPV
*Ectropis obliqua* NPV Strain A1	NC_008586	EcobNPV
*Euproctis pseudoconspersa* NPV	NC_012639	EupsNPV
*Helicoverpa armigera* MNPV	NC_011615	HaMNPV
*Helicoverpa armigera* NPV Isolate Australia	JN584482	HaNPV Aus
*Helicoverpa armigera* NPV Strain C1	NC_003094	HaNPV C1
*Helicoverpa armigera* NPV Strain G4	NC_002654	HaNPV G4
*Helicoverpa armigera* SNPV Strain NNg1	NC_011354	HaSNPV
*Helicoverpa zea* NPV	NC_003349	HezeNPV
*Hemileuca* sp. NPV	NC_021923	HespNPV
*Leucania separata* NPV Strain AH1	NC_008348	LeseNPV
*Lymantria dispar* MNPV	NC_001973	LdMNPV
*Lymantria xylina* MNPV	NC_013953	LyxyMNPV
*Mamestra brassicae* MNPV Isolate Chb1	JX138237	MabrMNPV Chb1
*Mamestra brassicae* MNPV Isolate K1	NC_023681	MabrMNPV K1
*Mamestra configurata* NPV Strain 90-2	NC_003529	MacoNPV 90 2
*Mamestra configurata* NPV Strain A90-4	AF539999	MacoNPV A90 4
*Mamestra configurata* NPV Strain B	NC_004117	MacoNPV B
*Orgyia leucostigma* NPV IsolateCFS77	NC_010276	OrleNPV
*Spodoptera exigua* MNPV	NC_002169	SeMNPV
*Spodoptera frugiperda* MNPV Isolate 3AP2	NC_009011	SfMNPV 3AP2
*Spodoptera frugiperda* MNPV Isolate Nicaraguan	HM595733	SfMNPV Nic
*Spodoptera frugiperda* MNPV Isolate Nicaraguan DefG	JF899325	SfMNPV NicG
*Spodoptera frugiperda* MNPV Strain 19	EU258200	SfMNPV 19
*Spodoptera littoralis* NPV Isolate AN1956	JX454574	SpltNPV AN1956
*Spodoptera litura* II MNPV	NC_011616	SpliMNPV II
*Spodoptera litura* MNPV Strain G2	NC_003102	SpliMNPV G2
*Trichoplusia ni* SNPV	NC_007383	TnSNPV
*Adoxophyes orana* GV	NC_005038	AdorGV
*Agrotis segetum* GV	NC_005839	AgseGV
*Choristoneura occidentalis* GV	NC_008168	ChocGV
*Clostera anastomosis* GV Strain Henan	NC_022646	CalGV
*Cryptophlebia leucotreta* GV	NC_005068	CrleGV
*Cydia pomonella* GV	NC_002816	CpGV
*Epinotia aporema* GV	NC_018875	EpapGV
*Helicoverpa armigera* GV	NC_010240	HearGV
*Phthorimaea operculella* GV	NC_004062	PhopGV
*Pseudaletia unipuncta* GV Strain Hawaiin	NC_013772	PsunGV
*Pieris rapae* GV	NC_013797	PiraGV
*Plutella xylostella* GV	NC_002593	PlxyGV
*Spodoptera litura* GV Strain K1	NC_009503	SpliGV
*Xestia c-nigrum* GV	NC_002331	XecnGV
*Neodiprion abietis* NPV	DQ317692	NeabNPV
*Neodiprion lecontei* NPV	NC_005906	NeleNPV
*Neodiprion sertifer* NPV	NC_005905	NeseNPV
*Culex nigripalpus* NPV	NC_003084	CuniNPV

### 2.4. Protein Synteny

BlastP analysis of SfGV VG008 proteome against the closest betabaculovirus proteomes (HearGV, PsunGV, SpliGV and XecnGV) were carried out using 0.001 as expected value (e-value) threshold. Once the corresponding orthologous proteins were detected, each SfGV VG008 protein was aligned against its ortholog in a pairwise fashion manner using ClustalW2 program and Gonnet 250 matrix of conservative changes [46]. Identity and similarity percentages were calculated for each alignment. The protein synteny graphs were generated using personal routines (P.D. Ghiringhelli, 2014, unpublished) with a color scale and similarity cut offs indicated in the corresponding figure. The length of the upper and lower prism sides is proportional to the length of the respective polypeptides.

### 2.5. Non-Coding Region Analyses

#### 2.5.1. Homologous Regions (*hrs*)

Canonical nucleotide decamers previously described in *hrs* of HearGV, PsunGV and XecnGV were used as computational probes to search the corresponding *hrs* in the SfGV VG008 genome. All non-coding regions containing repeats similar to probes were recovered and manually inspected. For each selected region, the secondary DNA structure prediction of the main sequence was obtained using the Mfold server of Michael Zuker website [47] and using RNADraw program [48]. In order to construct sequences logos, multiple alignments of palindromes were performed using ClustalX algorithm [41,49] with default parameters, and then sequences logos were obtained using the WebLogo server [50].

#### 2.5.2. A + T-Rich Regions

A + T-content was profiled using a partially overlapped sliding window strategy (window = 500 nucleotides, displacement = 50 nucleotides) (P.D. Ghiringhelli, 2012, unpublished). Relationships between each point and genomic average A + T-content were obtained and peaks of 1.12 or above were considered as A + T-rich regions.

### 2.6. Analyses of Genes Putatively Derived from Horizontal Transfer

#### 2.6.1. BlastP Relationships

First, a general baculoviral proteome database (GBPD) was constructed and BlastP searches using the SfGV VG008 proteome against GBPD were carried out (e-value threshold = 0.001). Candidate proteins acquired by horizontal transfer were selected. Then, individual specific protein databases (ISPDs) containing similar proteins of all related species were constructed to determine the relationships among selected species. Then, relaxed BlastP (e-value = 0.01) using individual proteins against the corresponding ISPD as a query was performed. Finally, the minimum e-values that showed reciprocity between pairs of viruses were selected and illustrated, involving all related viral species in network graphs.

#### 2.6.2. Recombination Analyses

In order to detect potential recombination events partial genome sequence of SfGV VG008 comprising the recombinant candidate genes (SfGV VG008 ORFs 059/099) and flanking regions was compared with the corresponding sequence in other baculoviruses (HearGV, PsunGV, XecnGV and SpltNPV II or SfMNPV) by running two alternative methods. In the first one (P.D. Ghiringhelli, 2008, unpublished), alignments were carried out with the ClustalX program (default parameters) [40,41,49] between sequence pairs, always involving the putative recombinant candidate from SfGV VG008 and one of the other sequences. The relative similarities were calculated using the ClustalX consensus symbol (* and blank space) as the input sequence, in an overlapping windows-based strategy. Arbitrary values of +1 for identical (*) and −0.5 for non- identical (blank spaces) residues were used. The sum of assigned values for each residue in each window (35 nucleotides) was divided by the window width and allotted to the central position to generate the plots. Profiles were drawn and analyzed with the aim of detecting crosspoints between them. In order to find a good relation between graph complexity and crosspoint detection sensitivity, different windows lengths were scanned. The second method was the bootscan analysis available in the Simplot program (version 3.5.1) [51,52] using the following parameters: (Window: 500 residues; Step: 50 residues; Gaps strip: on; Replicates: 100; Model: Kimura 2-parameters; Transition and transversion ratio: 2.0; Phylogenetic method: Neighbor-Joining). The breakpoints were estimated.

### 2.7. Characterization of SfGV VG008 ORFs 047/059/089/099

To determine the nature of SfGV VG008 ORF047, ORF059, ORF089 and ORF099 theoretical proteins, different bioinformatics tools were used. Hydrophobicity profiles were constructed using a sliding windows strategy (window = 17 amino acids; sliding 1 residue each time) and *ad hoc* program (P.D. Ghiringhelli, 2004, unpublished). Several hydrophobicity scales were assayed [53,54,55]. Signal peptide presence or absence was assessed by using SignalP [56]. Putative functions were evaluated using the HHpred server [57]. Secondary and tertiary structures were predicted using the LOcal MEta-Threading-Server [58], and the I-TASSER server [59] or the QUARK server [60]. Finally, the assessment of closest neighbors was carried out through phylogenetic inference of related sequence collections using MEGA program [44,45] with the following parameters: Method = Neighbor-Joining; Bootstrap with 500 replicates; gap/ Missing data = pairwise deletion; Model = Amino (Dayhoff Matrix); patterns among sites = Same (Homogeneous); rates among sites = Uniform Rates.

## 3. Results and Discussion

### 3.1. Genome of SfGV VG008 and Gene Content

The genome of SfGV VG008 (GenBank: KM371112) was covered 20 times and consists of 140,913 bp showing 53.8% of A + T content, a value very close to the lowest one estimated for betabaculovirus members which range between 53.2% in *Clostera anastomosis* GV and 67.5% in *Cryptophlebia leucotreta* GV [25]. However, no correlation was found between these data and virus biological properties impeding make predictions about features such as host range, pathogenicity or virulence [10].

**Figure 1:**
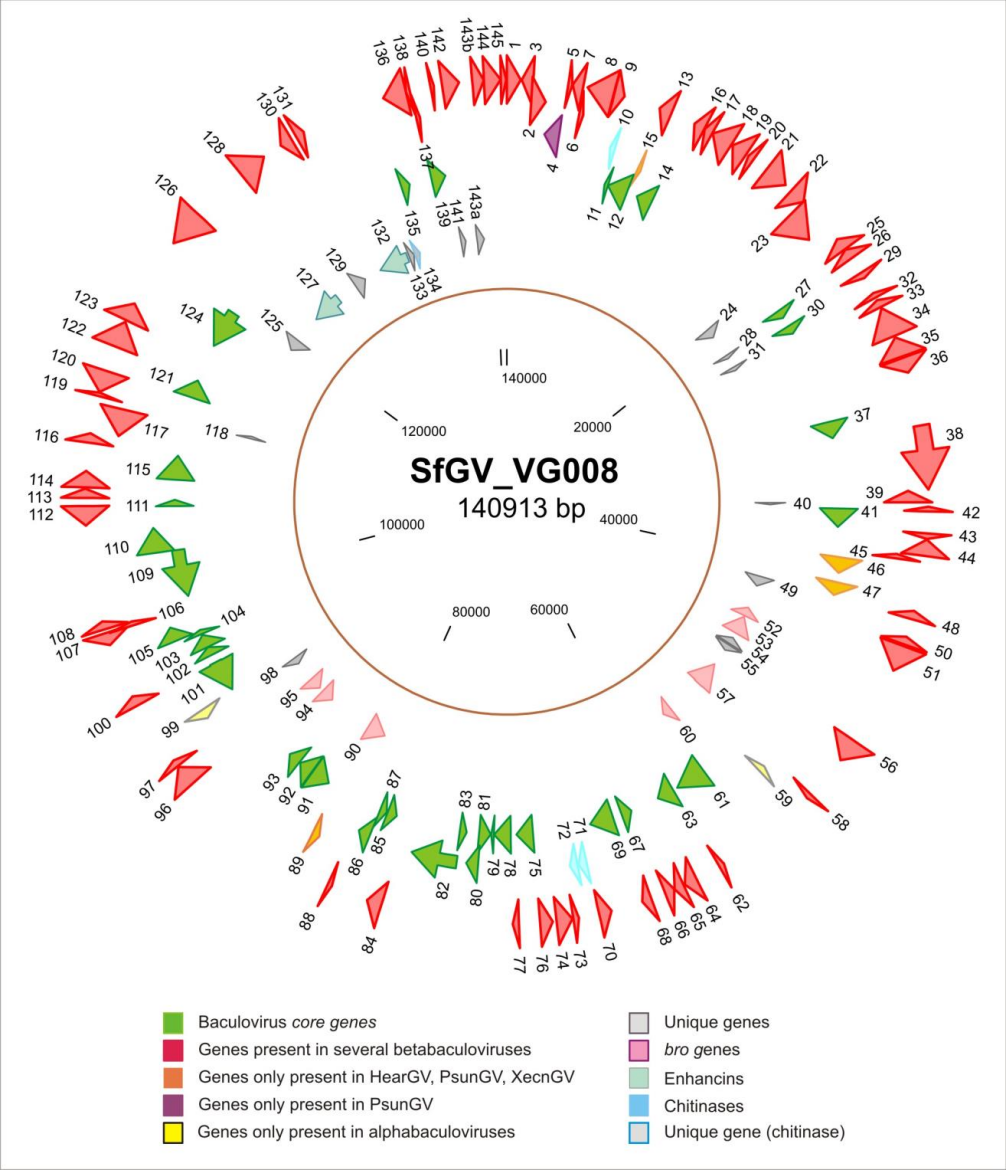
Circular map of the SfGV VG008 genome. The illustration shows all predicted SfGV ORFs (1–145 including 143a and 143b) and their transcription direction (with respect to *granulin*) indicated as arrows above a line that represent the genome (140,913 bp). The arrow colors represent different features such as presence of homologous sequences in other species of *Baculoviridae* and/or highlighting activities that include virulence factors (*enhancins* and *chitinases*). ORF’s numbers are indicated above or below the arrows. The names of the genes (assigned by significantly similarity with sequences form other baculoviruses) can be seen in Table S1. The regions without arrows represent non-coding regions.

The SfGV VG0008 genome contains 146 putative ORFs, all encoding theoretical polypeptides with at least 50 amino acid lengths and considering a minimal sequence overlapping among flanking regions. In view of the above, the ORFeome would cover 95.6% of the whole nucleotide sequence. ORFs were consecutively numbered from the first nucleotide of the *granulin* start codon resulting in 82 encoding regions in the *granulin* polarity and other 64 in the opposite one. The identity of genes was established by Blast (Figure 1).

To extend the previous study, typical promoter motifs located up to 120 bp upstream to the initial ATG and similarity comparison among orthologous genes from Noctuidae isolates (HearGV, PsunGV, SpliGV and XecnGV) were analyzed (Table S1). Therefore, that, early CAKT initiator sequence (INR) [61] was found in 47 ORFs, including or not TATA-box. Late INR motif [62] was detected in 21 ORFs; other 59 showed both early and late elements and 2 had only a TATA-box. The remaining 17 ORFs do not have any of the mentioned motifs, but could be transcribed from other regulatory elements [63].

Similarity analysis with the reported proteomes of each other baculovirus revealed that 125 proteins are shared with the all other betabaculoviruses meanwhile 2 are only shared with HearGV, PsunGV, XecnGV and SpltNPV II (ORF047 and ORF089). Other results showed that 2 proteins are shared with alphabaculoviruses (ORF059 and ORF099), 14 are unique (ORF024, ORF029, ORF031, ORF040, ORF049, ORF054, ORF055, ORF098, ORF118, ORF125, ORF129, ORF133, ORF134 and ORF141) and 3 seems to be product of divergent copies of some SfGV VG008 genes (sets of paralogous: ORF046 and ORF047; ORF057 -named Bro c- with ORF052 –Bro a-, ORF053 –Bro b-, ORF060 –Bro d-, ORF090 –Bro e-, ORF094 –Bro f- and ORF095 –Bro g-; ORF143a and ORF143b). One unique protein (ORF134) has similarity with a non-baculoviral *chitinase*-2c. In particular, the genome analysis revealed that SfGV VG008 would encode virulence factors associated to the enhancement of insecticidal activity [15]. Thus, this betabaculovirus contains four *chitinases* (ORFs 010, 071, 072, 134) and two *enhancins* (ORFs 127 and 132). Reviewing the 14 betabaculovirus genome analyses (papers cited in the introduction), only ChocGV, AgseGV, PsunGV, HearGV and XecnGV have genes encoding to enhancins (one for first two, three for the next and four ORFs for the others). Moreover, chitinase genes are present in two copies into ChocGV and EpapGV, and only one copy into CaLGV, ClanGV, CpGV, HearGV, PlxyGV and XecnGV genomes (CrleGV has a truncated chitinase gene). In this sense, SfGV VG008 is the betabaculovirus that would express more proteins associated to virulence.

### 3.2. Phylogenetic Inference for SfGV VG008

**Figure 2:**
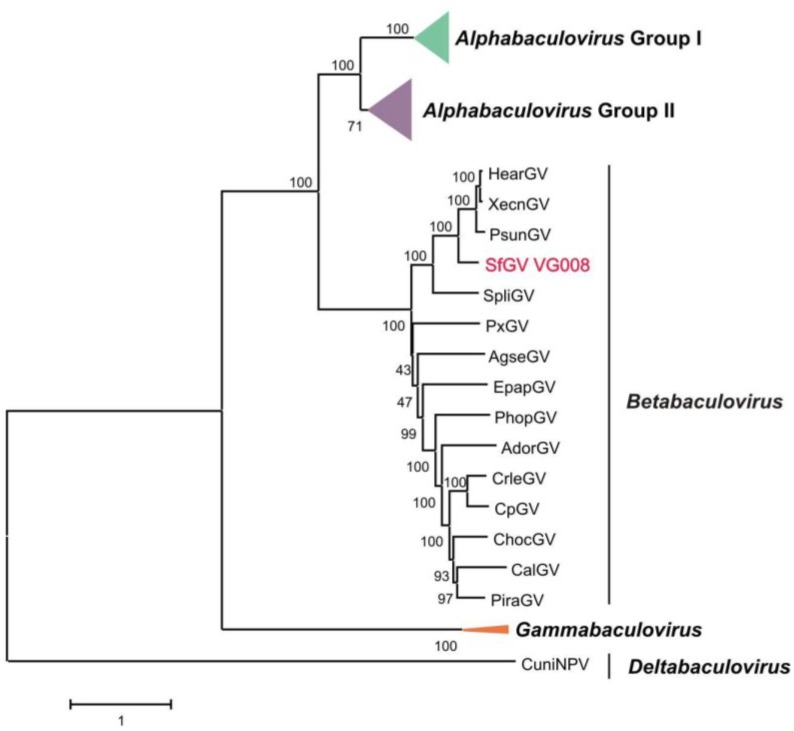
Phylogenetic inference for SfGV VG008. Cladogram based on a concatemer built with the 37 core proteins obtained from 74 baculoviral genomes. The phylogenetic tree was inferred using MEGA 6 program. The four *Baculoviridae* genera are indicated and in order to preserve space, *Alpha*- (Groups I and II) and *Gammabaculovirus* clades were collapsed.

As previously mentioned, ORFs encoding the 37 described core proteins for the *Baculoviridae* family [42] were found in the genome of SfGV VG008, covering the essential functions of: replication; transcription; cell cycle arrest and/or interaction with host proteins; viral packaging, assembly and release; and oral infectivity [64]. The phylogenetic analysis based on the 37 concatenated core proteins of 73 baculovirus genomes plus SfGV VG008 was performed (Figure 2).

The obtained cladogram reproduced the grouping of four genera recognized in the current classification of the family *Baculoviridae* [7]. As expected, SfGV VG008 isolated from Noctuidae insect, was a novel member of the *Betabaculovirus* genus grouping with HearGV, PsunGV, XecnGV and SpliGV. In previous reports it was observed that Noctuidae specific betabaculoviruses tend to be located in a separated group with respect to the members that infect the Tortricidae [8,65].

**Figure 3:**
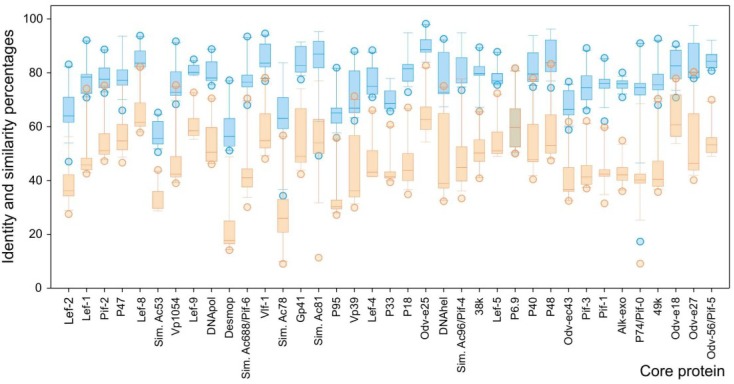
Identity and similarity analyses of SfGV VG008 core proteins. The box plot shows the amino acid identity (light orange) and similarity (light blue) percentages of the 37 core proteins present in SfGV VG008 respect to the orthologs located in the other betabaculoviruses. Core proteins names are the currently accepted (*Sim.* abbreviates *“similar to”*). The boundary of boxes closest to zero indicates the 25th percentile, a line within the box marks the median, and the boundary of the box farthest from zero indicates the 75th percentile. Error bars above and below boxes indicate the 90th and 10th percentiles, respectively. The filled circles indicate outlying points.

The theoretical proteins of core genes present in *Betabaculovirus* were compared in order to obtain pairwise identity and similarity values (Figure 3). According to this, the SfGV VG008 showed identity values ranges between 9% (SfGV ORF104 *vs.* EpapGV ORF043) and 83.3% (SfGV ORF103 *vs.* XecnGV ORF121) with a median of 48.4% (SfGV ORF078 *vs.* ClanGV ORF072). The equivalent study focused on similarity showed values ranges between 34.3% (SfGV ORF104 *vs.* CalGV ORF037) and 98.2% (SfGV ORF085 *vs.* HearGV ORF098) with a median of 77.6% (SfGV ORF037 *vs.* CpGV ORF047 and SfGV ORF069 *vs.* EpapGV ORF069).

The accepted function of each core protein and the ORF number according to genome annotations are detailed (Supplementary material Table S2). This study revealed that 37 core proteins are a set of factors strongly conserved into *Betabaculovirus* genus because they probably play the essentials roles needed to complete the virus cycle. So that, this set of ancestral sequences remains the best option for phylogenetic inference in *Baculoviridae*.

### 3.3. Genome Collinearity Analysis

**Figure 4:**
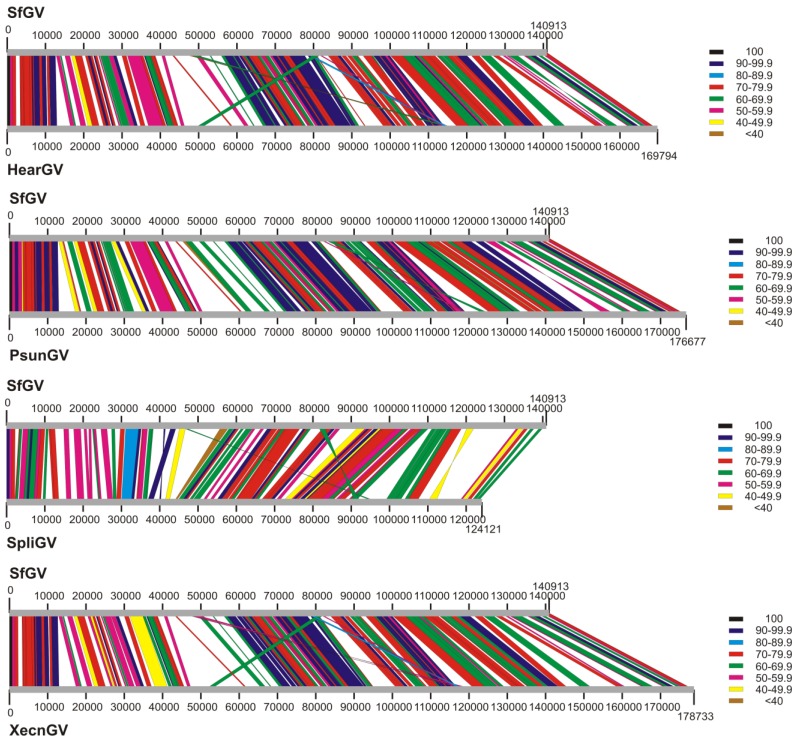
Protein synteny graphs. The illustration shows the comparison of gene collinearity based on genome physical positions and protein similarities between SfGV VG008 and each one of the most related betabaculoviruses (HearGV, PsunGV, SpliGV and XecnGV). The grey lines represent genomes and their lengths are proportional among them (bp scale). Colored lines between genomes (grey lines) relate homologous sequences indicating the percentage of similarity according to the key.

To characterize the genome organization of SfGV VG008, a gene collinearity study based on proteins with respect to the closely related betabaculoviruses (those isolated from Noctuides and which clustered in described phylogeny) was performed using synteny graphs (Figure 4, Supplementary Material Table S3). Thus, a great gene order correlation among the SfGV VG008, HearGV, PsunGV, SpliGV and XecnGV was observed, with some inversions and drifts. In general, the synteny maps are conserved among betabaculovirus species differing from alphabaculoviruses [21,25,28,30].

### 3.4. Homologous Regions (hrs) and A + T-Rich Regions

Baculovirus genomes have nucleotide sequence repeats known as homologous regions (*hrs*) that could act as replication starting points and/or as enhancers of transcription. Moreover, it is considered that these segments increase the genome plasticity and may mediate the intra and inter molecular recombination [63]. In addition to this, intergenic A + T-rich regions have been identified as putative non-*hr* origins of replication [66,67]. Considering the importance of this kind of sequences, the genome of SfGV VG008 was analyzed. Particularly, it contains 8 *hrs* [*hr*-1 (1 repeats), *hr*-2 (7 repeats), *hr*-3 (8 repeats), *hr*-4 (5 repeats), *hr*-5 (2 repeats), *hr*-6 (4 repeats), *hr*-7 (6 repeats) and *hr*-8 (1 repeats)]. Reviewing closest betabaculovirus genomes, 9 *hrs* has been described in HearGV [*hr*-1 (5 repeats), *hr*-2 (4 repeats), *hr*-3 (4 repeats), *hr*-4 (7 repeats), *hr*-5 (2 repeats), *hr*-5a (1 repeat), *hr*-6 (4 repeats), *hr*-7 (3 repeats) and *hr*-8 (4 repeats)], 9 in PsunGV [*hr*-1 (5 repeats), *hr*-2 (3 repeats), *hr*-3 (4 repeats), *hr*-4 (5 repeats), *hr*-5 (2 repeats), *hr*-5a (1 repeat), *hr*-6 (3 repeats), *hr*-7 (4 repeats) and *hr*-8 (3 repeats)], and 9 in XecnGV genomes [*hr*-1 (5 repeats), *hr*-2 (5 repeats), *hr*-3 (4 repeats), *hr*-4 (6 repeats), *hr*-5 (3 repeats), *hr*-5a (1 repeat), *hr*-6 (4 repeats), *hr*-7 (5 repeats) and *hr*-8 (4 repeats)].

**Figure 5:**
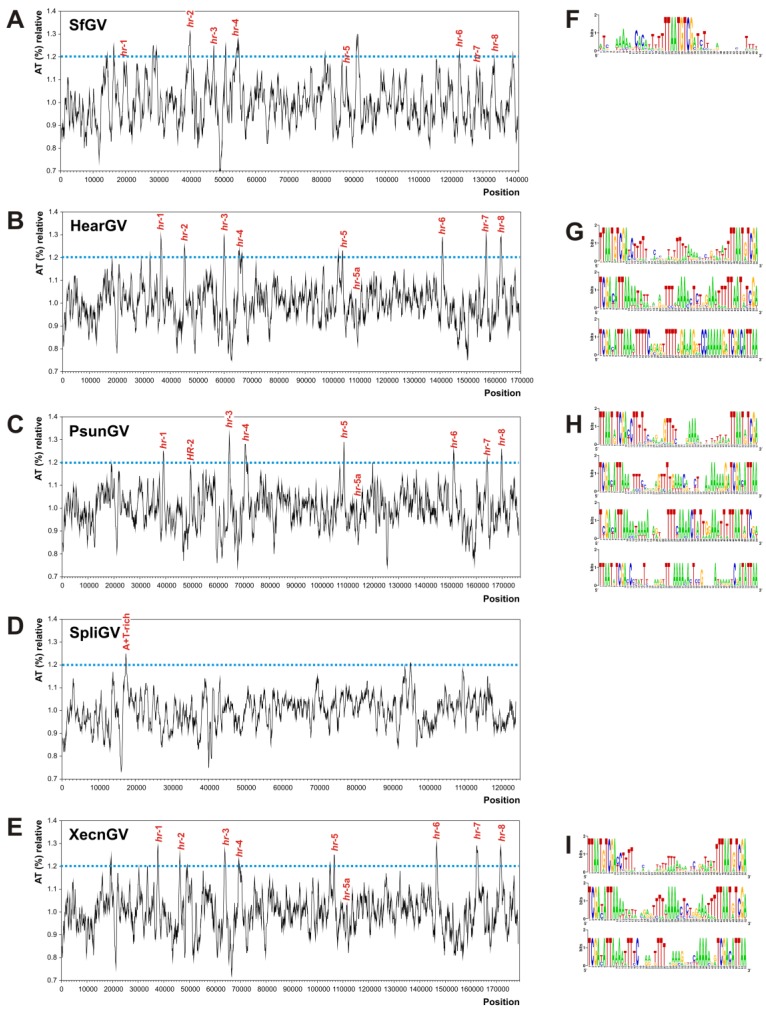
A + T-rich profiles and characterization of hrs. The plot graphs represent the profiles of A + T-content indicated as relative percentages along some betabaculovirus genomes. Blue lines show the cut off used to define the A + T-rich regions. The *hrs* sequences are indicated in the corresponding positions of each genome (for SpliGV, *hrs* were not reported). (**A**) A + T profile of SfGV VG008 genome; (**B**) A + T profile of HearGV genome; (**C**) A + T profile of PsunGV genome; (**D**) A + T profile of SpliGV genome; (**E**) A + T profile of XecnGV genome; (**F**) Sequence logo of the motif of *hrs* from SfGV VG008; (**G**). Sequence logo of the three main motifs of *hrs* from HearGV; (**H)** Sequence logo of the four main motifs of *hrs* from PsunGV; (**I)** Sequence logo of the three main motifs of *hrs* from XecnGV.

A deep analysis showed that repeats are stretches of 40–48 nucleotides length, in which the 10 bp at each end are perfect direct or inverted sequences, and only in few cases one of the flank is an imperfect repetition. All *hrs* were found within A + T-rich regions (Figure 5). It is important to note that SpliGV contains one A + T-rich region but sequences like hrs were not found [68]. This observation suggests that hrs may not be essentials in baculovirus cycle but surely their presence positively contribute in the other important processes previously mentioned, such as gene acquisition, genome replication or transcription enhancement.

HearGV, PsunGV and XcenGV have *hrs* of variable lenght (50–58 nt) where the first and last 10 nucleotides are perfect or imperfect copies (direct or inverted) of a core oligonucleotide [TTAAT(G/A)TGCA] which flank variable regions (30–38 nt) rich in AT content. Besides, it is possible to detect, by clustering analyses, 3 *hr* variants for HearGV and XcenGV, and 4 for PsunGV. In contrast, SfGV VG008 has other organization because each *hr* contains one sequence unit (TTAATGTGC) located into A + T rich regions of about 50 nucleotides (Table S4).

It is known that these regions may differ in location within genomes, number of copies and nucleotide sequences between different baculovirus species; however, their generalized distribution suggest that functions are conserved [66,69]. In general, the non-coding regions of baculovirus genomes represent less than 10% of whole sequence and this fraction usually contains the *hrs* [67]. In this sense, six *hrs* of SfGV VG008 were found into non-coding regions [*hr-*2 (39,744–40,073 bp), *hr-*3 (46,924–47,327 bp), *hr-*4 (53,816–54,064 bp), *hr-*5 (87,869–87,960 bp), *hr-*6 (122,550 -122,734 bp) and *hr-*8 (133,555–133,603 bp)] and the other two *hrs* [*hr-*1 (19,328–19,376 bp) and *hr-*7 (128,872–129,176 bp)] were located in zones that show a minimal overlapping with encoding regions (SfGV VG008 ORF 024 for *hr-*1 and ORFs 130/131 for *hr-*7).

### 3.5. Analyses of Genes Putatively Derived from Horizontal Transfer

Core genes in *Baculoviridae* are vertically transferred from the last virus common ancestor [42]. In contrast, other genes were after incorporated in particular species by horizontal transmission by processes that include recombination and transposition events. In SfGV VG008 three genes (ORF046, ORF047 and ORF089) are only shared with some betabaculoviruses and some alphabaculoviruses of Group II. Similarly, 2 other genes (ORF059 and ORF099) are shared only with alphabaculoviruses suggesting origins by horizontal transfer.

**Figure 6:**
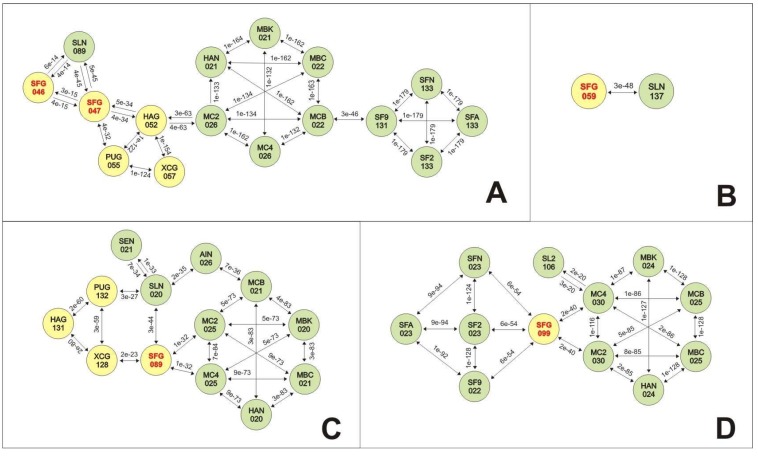
Protein relationships for SfGV VG008 ORFs 046/047/059/089/099. The relationships among some ORFs of SfGV VG008 and their orthologous genes contained in other viruses were calculated by BlastP. The illustration shows the related baculovirus species indicating a name abbreviation for each one (three letters for species and ORF number) into filled circles (yellow for betabaculoviruses and green for Group II alphabaculoviruses). The BlastP e-value between pairs of species is indicated above each arrow. (**A**) Protein relationships for SfGV VG008 ORFs 046/047; (**B**) Protein relationships for SfGV VG008 ORF059; (**C)** Protein relationships for SfGV VG008 ORF089; (**D)** Protein relationships for SfGV VG008 ORF099. AIN: AgipNPV; HAG: HearGV; HAN: HearMNPV; MC2: MacoNPV 90-2; MC4: MacoNPV A90-4; MBC: MabrNPV CHb1; MBK: MabrNPV K1; MCB: MacoNPV B; PUG: PsunGV; SEN: SeMNPV; SF2: SfMNPV 3AP2; SF9: SfMNPV 19; SFA: SfMNPV Nic DefG; SFG: SfGV VG 008 (in red letters); SFN: SfMNPV Nic; SL2: SpliNPV G2; SLN: SpltNPV II; XCG: XecnGV.

In order to add information that clarify this supposition, different studies were performed. First, all species were related in a network where the minimum BlastP e-values obtained for pairs of viruses were selected (Figure 6). It is important to note that the reciprocal e-values were not always coincident because some proteins have different sizes.

#### 3.5.1. SfGV VG008 ORFs 046/047/089

Detailed analysis of SfGV VG008 ORF047 with respect to the most similar proteins present in HearGV, PsunGV, XecnGV and SpltNPV II shows identity/similarity values of 27.5%/61.2%, 29.1%/61.1%, 27.4%/63.3% and 37.6%/60.9%, respectively. Although the highest identity value is between SfGV VG008 and SpltNPV II, the origin of the gene that encodes this protein is unclear because the significance of differences in the similarity values between SfGV VG008 ORF047 and the other proteins are doubtful. However, the BlastP searches for similar proteins in the corresponding ISPD shows a stronger relationship between SfGV VG008 ORF047 and SpltNPV II ORF089 than can be found between SfGV VG008 ORF047 and the similar proteins of the most related betabaculoviruses. This relationship could be derived from an ancient acquisition and parallel evolution.

The analysis of SfGV VG008 ORF089 with the similar proteins from HearGV, PsunGV, XecnGV, SpltNPV II, MacoNPV 90-2 and MacoNPV A90-4 shows identity/similarity values of 31.4%/67.9%, 31.7%/67.7%, 33.3%/69.8%, 48.5%/79.1%, 44.7%/66.5% and 44.7%/66.5%, respectively. The highest values are those obtained when the comparison was with SpltNPV II, although the differences in identity between SfGV VG008 ORF089 and the corresponding proteins of SpltNPV II, MacoNPV 90-2 and MacoNPV A90-4 are minimal. However, the BlastP searches for similar proteins in the corresponding ISPD shows a higher relationship between SfGV VG008 ORF089 and SpltNPV II ORF020 than can be found between SfGV VG008 ORF089 and the similar proteins of the most related betabaculoviruses, MacoNPV 90-2 or MacoNPV A90-4. According to this, at least two different evolutive scenarios could have occurred. In the first one, SpltNPV II or MacoNPV 90-2 or MacoNPV A90-4 has acted as the source of the gene in independent events of horizontal transfer to betabaculoviruses; differences in the ancestrality of this process could explain the present identity/similarity values before showed. In the second one, SpltNPV II or MacoNPV 90-2 or MacoNPV A90-4 has acted as the donor species in a horizontal transfer event, which has occurred with a hypothetical common ancestor of HearGV, PsunGV, SfGV and XecnGV. The differences in the speciation and divergency times could explain the present identity/similarity values.

#### 3.5.2. SfGV VG008 ORFs 059/099

The situation is most clear in the case of proteins SfGV VG008 ORF059 and SfGV VG008 ORF099 because they are only shared with alphabaculoviruses of Group II. SfGV VG008 ORF059 is only similar to the protein encoded in the ORF137 of SpltNPV II, whereas SfGV VG008 ORF099 is similar to the proteins encoded in: HearMNPV ORF024, MacoNPV 90-2 ORF030, MacoNPV A90-4 ORF030, MacoNPV B ORF025, MabrMNPV Chb1 ORF025, MabrMNPV K1 ORF024, SfMNPV 3AP2 ORF023, SfMNPV 19 ORF022, SfMNPV Nic ORF023, SfMNPV Isolate Nic DefG ORF023 and SpltNPV G2 ORF106. The highest identity/similarity values is between SfGV VG008 ORF099 and ORF023 of the SfMNPV isolates (ORF022 in SfMNPV 19), although the similarity between SfGV VG008 ORF099 and the corresponding proteins of SfMNPV or MacoNPV isolates converged to nearest values. However, the BlastP searches for similar proteins in the corresponding ISPD shows a higher relationship among SfGV VG008 ORF099 and similar proteins of SfMNPV Nic, SfMNPV 3AP2 and SfMNPV 19 isolates than can be found among SfGV VG008 ORF099 and the similar proteins of the other related alphabaculoviruses of Group II. In both cases, the obtained results suggest the occurrence of independent horizontal transfer events during the evolution.

**Figure 7:**
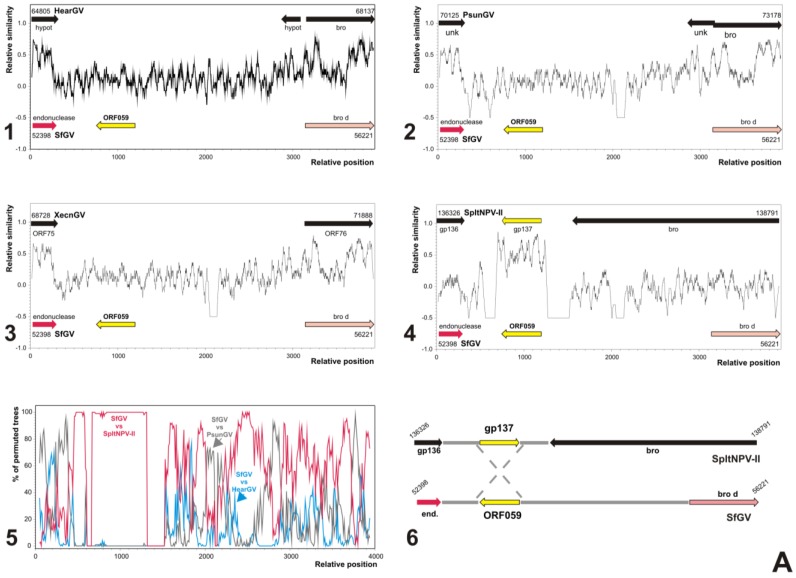

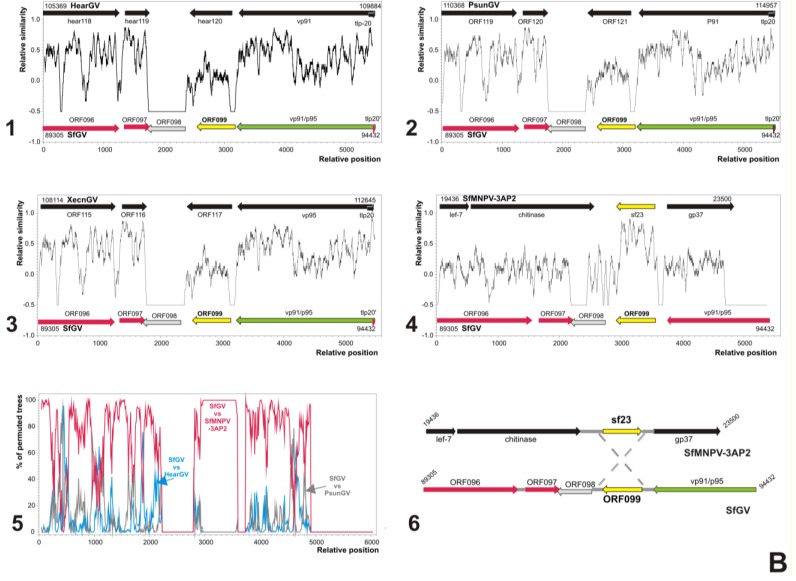
Origin by horizontal transfer of SfGV VG008 ORFs 059 and 099. Similarity plots and bootscanning analysis for possible recombination processes between the ancestors of SfGV VG008 and other baculoviruses are studied. The genome regions considered are those containing SfGV VG008 ORFs 059 and 099 (about 3900 bp and 5600 bp, respectively). In all cases, colored arrows according to the key indicated in Figure 1 represent ORFs of SfGV VG008. In contrast, ORFs of the other baculoviruses are represented as black arrows excepting homologous genes to SfGV VG008 ORFs 059 and 099 (yellow arrows). All the genome positions in the studies are indicated at the beginning and the end of considered regions (bp scale). The similarity plots are indicated in black, and in the bootscanning analyses different colors are used being referenced into the graphs. (**A**) Possible origin of SfGV GV008 ORF059. 1. Similarity plot between SfGV VG008 and HearGV; 2. Similarity plot between SfGV VG008 and PsunGV; 3. Similarity plot between SfGV VG008 and XecnGV; 4. Similarity plot between SfGV VG008 and SpltNPV II; 5. Bootscanning using SfGV VG008, HearGV, PsunGV, XecnGV and SpltNPV II; 6. Partial genomic maps of SpltNPV II and SfGV VG008, and putative recombination event; (**B**) Possible origin of SfGV GV008 ORF099. 1. Similarity plot between SfGV VG008 and HearGV; 2. Similarity plot between SfGV VG008 and PsunGV; 3. Similarity plot between SfGV VG008 and XecnGV; 4. Similarity plot between SfGV VG008 and SfMNPV-3AP2; 5. Bootscanning using SfGV VG008, HearGV, PsunGV, XecnGV and SfMNPV-3AP2; 6. Partial genomic maps of SfMNPV-3AP2 and SfGV VG008, and putative recombination event.

In order to explore the recombination hypothesis for SfGV VG008 ORF059 and ORF099 two approaches were used: relative similarity and bootscaning analyses (Figure 7). Thus, the results obtained with mentioned studies for both genes supported the hypothesis, which showed the occurrence of putative recombination events. Particularly, studies of relative similarity showed that any betabaculoviruses from Noctuide insects (HearGV, PsunGV and XcenGV) present homologous sequences to SfGV VG008 ORF059. In contrast, when the comparison was with SpltNPV II the relative similarity was significantly high with SpltNPV II ORF137. In fact, this observation was confirmed with high percentage of permuted trees by bootscanning plot against previously mentioned betabaculoviruses and SpltNPV II suggesting a recombination event involving two different virus species.

In the case of SfGV VG008 ORF099 similar results were observed, although the relative similarities and the bootscanning plots showed that the sequence donor probably be the SfMNPV genome or some relative ancestors. Besides, it is important to note that upstream to recombination site there is a SfGV GV008 unique ORF (ORF098). Both recombination events seem to have occurred in non-coding regions by insertion or by replacement of a stretch of non-coding sequence. Importantly, very close to SfGV VG008 ORF59 (53,537–53,124 bp) is the *hr-*4 (53,816–54,064 bp) that could be associated with the recombination event as previously reported [70].

Recombination may serve as a mechanism for baculoviruses to rapidly obtain the genetic variation required for survival in a non-static environment without the potential loss of viability that may occur with a high mutation rate [70]. These events in the *Baculoviridae* family have been observed between different viral species (heterologous recombination) involving genomic DNA exchange in natural co-infections and in cell cultures exposed to different viruses, and between very close related viruses (homologous recombination) [70,71].

It has been shown that recombination events allow some baculovirus expand its host range, as is the case between AcMNPV and BmNPV [72]. This feature has been studied as an alternative to extend the usefulness of baculovirus as biotechnological tool or as biopesticides [73].

Is important to note that the SfGV VG008 isolate was found in *S. frugiperda* larvae collected in the field, which presented a natural co-infection with a Colombian isolate of SfMNPV (SfCOL). In this sense, events of intra- and inter-specific recombination during co-infection in the same host have been described for some isolates of CrleGV and CpGV [74]. These processes have also been observed in alphabaculoviruses, where heterologous recombination between isolates of AcMNPV and RoMNPV in natural populations of *Galleria mellonella* (Linnaeus, 1758) (Lepidoptera: Pyralidae) was reported [75].

Similar to this study, ORFs derived from possible independent recombination events with SeMNPV and/or SpltMNPV has been reported for Colombian SfMNPV isolate [4]. These sequences did not present homologies with the other SfMNPV isolates. Specifically, the ORFs 4 and 5 share high similarities with ORFs splt20 and splt21 of the SpltMNPV genome respectively, and with ORFs se21 and se22 + se23 of SeMNPV genome. In contrast, Colombian SfMNPV lacked the sf23 ORF (unknown function) which is found in the other reported SfMNPV genomes [4].

### 3.6. Characterization of Proteins Encoded by SfGV VG008 ORFs 047/059/089/099

**Figure 8:**
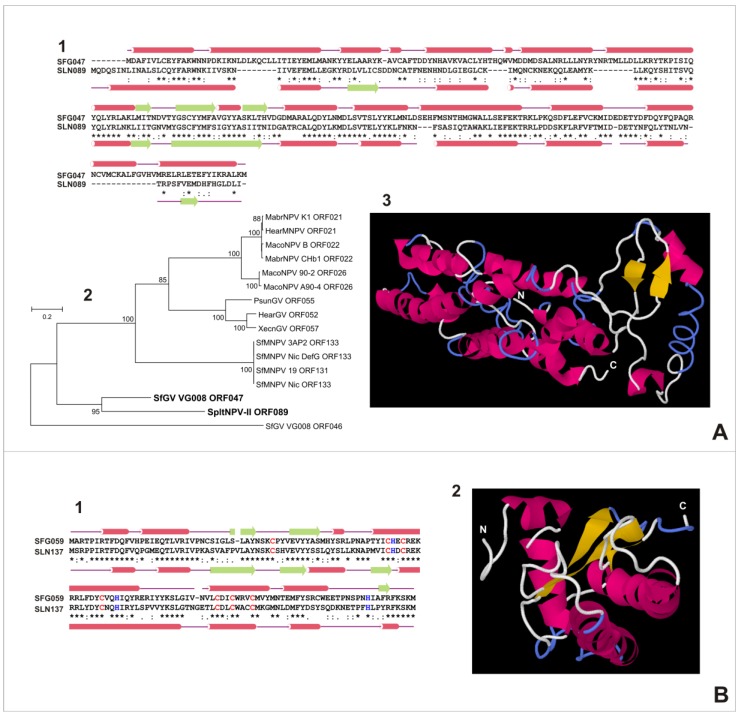

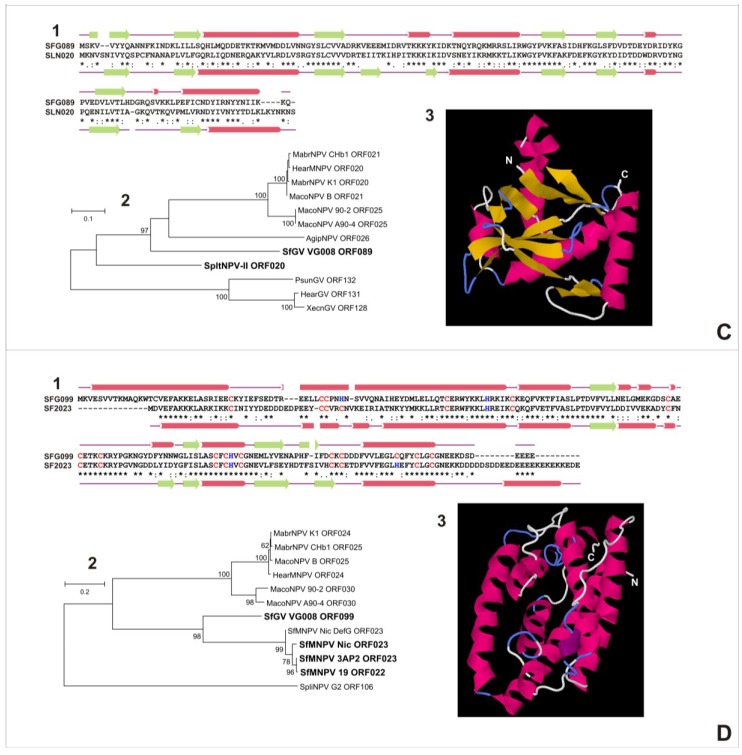
Characterization of proteins derived from SfGV VG008 ORFs 047/059/089/099. The theoretical proteins encoded by SfGV VG008 ORFs 047 (panel **A**), 059 (panel **B**), 089 (panel **C**) and 099 (panel **D**) were analyzed. Pairwise sequence alignment between previously mentioned proteins from SfGV and their closest homologs (highlighting the predicted secondary structures) are shown (A1; B1; C1; D1). Alpha helices are represented as red cylinders and beta sheets as green arrows. Particular amino acids are also indicated (cysteins in red and histidines in blue) for SfGV VG008 ORFs 059 and 099 indicating conserved residues putatively involved in a ring-finger motif. Besides, the evolutionary histories were inferred using the protein sequence collection derived from previous BlastP relationship analyses (Figure 6). Cladograms contain on the branches the percentage of replicated trees in which the associated taxa clustered in the bootstrap test, only indicating those values greater than 60% (A2; C2; D2). In all cases, the most related sequences are highlighted in bold letter. Moreover, 3D structures were predicted using LOMETS (A3; C3) or QUARQS (B3) or I-TASSER (C3) servers. SF2: SfMNPV 3AP2; SFG: SfGV VG 008; SLN: SpltNPV II.

Considering the previous results, the genes obtained by horizontal transfer were particularly analyzed in terms of protein structure and phylogenetic relationships with other baculovirus species (Figure 8).

SfGV VG008 ORF047 protein consists of 263 residues, with 33 negatively charged (Asp + Glu) and 38 positively charged (31 Arg + Lys, and 7 His) amino acids. Based on sequence, the molecular weight is 31,401.9 kDa and its net charge is +1.5. The hydrophobicity profile suggests that is a soluble protein with an average hydrophobicity of −0.58. Secondary structure prediction using the LOMETS server shows that 216 residues (82.1%) are distributed in at least 12 alpha helices (74.0%) and 3 beta sheets (6.1%), while the other ones are part of loops or turns. According to previously mentioned material, the same software predicted that SfGV VG008 ORF047 is a globular protein. On the other hand, the polypeptide of SfGV and the SpltNPV II ORF089 protein share 64.6% of the three-state secondary structure motifs. To assess the hypotheses that SpltNPV II ORF089 protein is the closest sequence as was previously determined by the BlastP relationship, an evolutionary history of all related baculoviral proteins was inferred using the Neighbor-Joining method. According to this, the phylogenetic tree showed a strong correlation with the above adding evidence for this assumption. Moreover, HHpred software did not identify any specific domain but showed some similarity with proteins that participate in the transcription process, such as the Ribbon-helix-helix motif acting in regulation of transcription DNA-templated, late transcription factor VLTF-2 (also called Poxvirus trans-activator protein A1), and transcription initiation factor IIA (gamma chain in *Homo sapiens* and small chain in *Saccharomyces cerevisiae*). Anyway, the biological role of SfGV VG008 ORF047 in the transcription process requires experimental confirmation.

SfGV VG008 ORF089 protein consists of 156 residues, with 24 negatively charged (Asp + Glu) and 31 positively charged (28 Arg + Lys, and 3 His) amino acids. Based on the sequence, the molecular weight is 18,648.8 kDa and its net charge is +5.5. The hydrophobicity profile suggests that it is a soluble protein with an average hydrophobicity of −1.06. Secondary structure prediction using the QUARK server shows that 91 residues (58.3%) are distributed in at least 4 alpha helices (37.8%) and 6 beta sheets (20.5%), while the other ones are part of loops or turns. Besides, this software predicted a globular topology. On the other hand, SfGV VG008 ORF089 and SpltNPV II ORF020 proteins share 81.0% of the three-state secondary structure motifs, result that was supported by the phylogenetic inference. In SpltNPV II ORF020 protein HHpred identified a phosphatase domain comprised between the amino acids 35 and 96, similar to Polynucleotide kinase 3 phosphatase of *Schizosaccaromyces pombe* (PNK1) [76], which play a role in the repair of single breaks in DNA induced by several DNA-damaging agents. HHpred finding on SfGV VG008 ORF089 protein and local sequence similarity studies performed for both proteins supported the previous function assignment and detect the phosphatase domain between residues 33 to 94. Nevertheless, if SfGV VG008 ORF089 acts in DNA repairing processes requires further confirmation.

The SfGV VG008 ORF059 protein consists of 137 residues, with 12 negatively charged (Asp + Glu) and 22 positively charged (17 Arg + Lys, and 5 His) amino acids. Based on the sequence, the molecular weight is 16,518.4 kDa and its net charge is +5.5. The hydrophobicity profile suggests that is a soluble protein with an average hydrophobicity of −0.71. Secondary structure prediction using the QUARK server shows that 91 residues (56.9%) are distributed in at least 4 alpha helices (48.2%) and 2 beta sheets (8.8%), while the other ones are part of loops or turns. Besides, that software predicted a globular topology. On the other hand, SfGV VG008 ORF059 and SpltNPV II ORF137 proteins share 66.9% of the three-state secondary structure motifs and due to both polypeptides are similar and unique within *Baculoviridae*, the assessment of which sequence is the phylogenetically closest was not necessary. HHpred identified the typical presence and distribution of Cys (C) and His (H) residues that occur in the Ring-finger family domains. Particularly, SfGV VG008 ORF059 protein contains 9 cysteines and 5 histidines while SpltNPV II ORF137 protein has 8 cysteines and 4 histidines. Besides, a high conservation in polypeptide position was detected by pairwise alignment (7 C and 3 H) constituting the following motif: x(39-40)-C-x(22)-C-H-x-C-x(9)-C-x(2)-H-x(18-19)-C-x(2)-C-x(3)-C-x(22)-H-x(9). It is possible that some of these residues could coordinate Zn^+2^ (e.g., Zinc-fingers) or other divalent cations. In most cases, Zinc-fingers are typical motifs distributed in DNA/RNA regulatory proteins whereas the coordination of heavy metals is often a characteristic of different metallothioneins. Any of these assumptions must be experimentally corroborated.

Finally, the SfGV VG008 ORF099 protein consists of 198 residues, with 37 negatively charged (Asp + Glu) and 28 positively charged (23 Arg + Lys, and 5 His) amino acids. Based on the sequence, the molecular weight is 23,055.3 kDa and its net charge is −11.5. The hydrophobicity profile suggests that is a soluble protein with an average hydrophobicity of −0.72. Secondary structure prediction using the I-TASSER server shows that 134 residues (67.7%) are distributed in at least 6 alpha helices (59.1%) and 3 beta sheets (8.6%), while the other ones are part of loops or turns. Besides, that software predicted a globular topology. On the other hand, SfGV VG008 ORF099 and SfMNPV 3AP2 ORF023 proteins share 70.9% of the three-state secondary structure motifs, a result that was supported by the phylogenetic inference. As occurred with the SfGV VG008 ORF099 protein, HHpred identified C and H residues distributed like Ring-finger domains. In particular, this protein contains 17 C and 5 H whereas SfMNPV 3AP2 ORF023 protein has 16 C and 5 H. Besides, a high conservation in polypeptide position was detected by pairwise alignment (15 C and 2 H) constituting the following motif: x(16-30)-C-x(14-16)-C-C-x(2)-[CH]-x(19-20)-C-x(7)-H-x(4)-C-x(30)-C-x(2)-C-x(3)-C-x(22)-C-x-C-H-x-C-x(15-16)-C-x-C-x(10)-[CH]-x(3)-C-x(2)-C-x(12-29). Similar to previous results, speculations can be made about the biological role of this polypeptide any of which will require experimental assays.

## 4. Concluding Remarks

This work presents the first genome analysis of a betabaculovirus from a *S. frugiperda*, named SfGV VG008, revealing that Noctuide insects are usually infected by phylogenetically closely related GVs. However, this virus contains 14 unique genes, including one encoding a putative chitinase protein. Additionally, the gene content shows that SfGV VG008 possesses encoding sequences for other virulence factors to insecticidal activity, such as 3 chitinases and 2 enhancins homologs to other baculoviruses. This observation offers the possibility to postulate it as enhancer factor in NPVs bio-insecticide formulations for *S. frugiperda*. Moreover, the SfGV VG008 genome analysis suggests that its ancestors acquired some genes by horizontal transfer from alpha- and betabaculoviruses. In view of the high number of unique genes, the evidence here observed about recombination events and the ability to co-infect a host with other baculovirus species, SfGV VG008 is an example of how the *Baculoviridae* family members maintain the diversity, improve their fitness, spread host range and assure their perpetuation in nature.

## Supplementary Files

Supplementary File 1

## References

[1] Clark P.L., Molina-Ochoa J., Martinelli S., Skoda S.R., Isenhour D.J., Lee D.J., Krumm J.T., Foster J.E. (2007). Population variation of the fall armyworm, *Spodoptera frugiperda*, in the western hemisphere. J. Insect Sci..

[2] Williams T., Goulson D., Caballero P., Cisneros J., Martínez A.M., Chapman J.W., Roman D.X., Cave R.D. (1999). Evaluation of a baculovirus bioinsecticide for small-scale maize growers in latin america. Biol. Control.

[3] Flores F. (2010). Manejo de Plagas en el Cultivo de Maíz.

[4] Barrera G., Simón O., Villamizar L., Williams T., Caballero P. (2011). *Spodoptera frugiperda* multiple nucleopolyhedrovirus as a potential biological insecticide: Genetic and phenotypic comparison of field isolates from colombia. Biol. Control.

[5] Lapied B., Pennetier C., Apaire-Marchais V., Licznar P., Corbel V. (2009). Innovative applications for insect viruses: Towards insecticide sensitization. Trends Biotechnol..

[6] Moscardi F., Souza M., Castro M., Moscardi M., Szewczyk B., Ahmad I., Ahmad F., Pichtel J. (2011). Baculovirus pesticides: Present state and future perspectives. Microbes and Microbial Technology.

[7] Herniou E., Arif B.M., Becnel J., Blissard G.W., Bonning B.C., Harrison R.L., Jehle J.A., Theilmann D., Vlak J.M., King A.M., Adams M.J., Carstens E.B., Lefkowitz E.J. (2012). Family *baculoviridae*. Virus Taxonomy—Classification and Nomenclature of Viruses: Ninth Report of the Internationalcommittee on Taxonomy of Viruses.

[8] Jehle J.A., Lange M., Wang H.L., Hu Z.H., Wang Y.J., Hauschild W. (2006). Molecular identification and phylogenetic analysis of baculoviruses from lepidoptera. Virology.

[9] Rohrmann G.F. Baculovirus Molecular Biology. http://www.ncbi.nlm.nih.gov/books/NBK114593/.

[10] Ferrelli M.L., Berretta M.F., Belaich M.N., Ghiringhelli P.D., Sciocco-Cap A., Romanowski V., Garcia M.L., Romanowsky V. (2012). The baculoviral genome. Viral Genomes—Molecular Structure, Diversity, Gene Expression Mechanisms and Host-Virus Interactions.

[11] Slack J., Arif B.M. (2007). The baculoviruses occlusion-derived virus: Virion structure and function. Adv. Virus Res..

[12] Ji X., Sutton G., Evans G., Axford D., Owen R., Stuart D.I. (2010). How baculovirus polyhedra fit square pegs into round holes to robustly package viruses. EMBO J..

[13] Jackes R.P., Maromorosch E., Sherman K. (1985). Stability of insect viruses in the environment. Viral Insecticides for Biological Control.

[14] Williams G.V., Faulkner P., Miller L. (1997). Cytological changes and viral morphogenesis during baculovirus infection. The Baculoviruses.

[15] Hoover K., Humphries M.A., Gendron A.R., Slavicek J.M. (2010). Impact of viral enhancin genes on potency of *lymantria dispar* multiple nucleopolyhedrovirus in *L. dispar* following disruption of the peritrophic matrix. J. Invertebr. Pathol..

[16] Mukawa S., Goto C. (2011). Enhancing effect of proteins derived from *Xestia c-nigrum* granulovirus on *Mamestra brassicae* nucleopolyhedrovirus infection in larvae of *Autographa nigrisigna* (lepidoptera: Noctuidae) on cabbage. Appl. Entomol. Zool..

[17] Gómez J.A., Barrera G., Guevara J., Villamizar L. (2010). Aislamiento y evaluación de un nucleopoliedrovirus colombiano de *Spodoptera frugiperda* para su control. Mem. XXXIII Congr. Nac. Control Biol..

[18] Cuartas P., Barrera G., Barreto E., Villamizar L. (2014). Characterisation of a colombian granulovirus *(Baculoviridae*: Betabaculovirus) isolated from *Spodoptera frugiperda* (lepidoptera: Noctuidae) larvae. Biocontrol. Sci. Technol..

[19] National Center for Biotechnology Information. http://www.ncbi.nlm.nih.gov.

[20] Escasa S.R., Lauzon H.A., Mathur A.C., Krell P.J., Arif B.M. (2006). Sequence analysis of the *Choristoneura occidentalis* granulovirus genome. J. Gen. Virol..

[21] Ferrelli M.L., Salvador R., Biedma M.E., Berretta M.F., Haase S., Sciocco-Cap A., Ghiringhelli P.D., Romanowski V. (2012). Genome of *Epinotia aporema* granulovirus (epapgv), a polyorganotropic fast killing betabaculovirus with a novel thymidylate kinase gene. BMC Genomics.

[22] Harrison R.L., Popham H.J.R. (2008). Genomic sequence analysis of a granulovirus isolated from the old world bollworm, *Helicoverpa armigera*. Virus Genes.

[23] Hashimoto Y., Hayakawa T., Ueno Y., Fujita T., Sano Y., Matsumoto T. (2000). Sequence analysis of the *Plutella xylostella* granulovirus genome. Virology.

[24] Hayakawa T., Ko R., Okano K., Seong S.-I., Goto C., Maeda S. (1999). Sequence analysis of the *Xestia c-nigrum* granulovirus genome. Virology.

[25] Lange M., Jehle J.A. (2003). The genome of the *Cryptophlebia leucotreta* granulovirus. Virology.

[26] Liang Z., Zhang X., Yin X., Cao S., Xu F. (2011). Genomic sequencing and analysis of *Clostera anachoreta* granulovirus. Arch. Virol..

[27] Liang Z., Zhang X., Yin X., Song X., Shao X., Wang L. (2013). Comparative analysis of the genomes of *Clostera anastomosis* (l) granulovirus and *Clostera anachoreta* granulovirus. Arch. Virol..

[28] Luque T., Finch R., Crook N., O’Reilly D.R., Winstanley D. (2001). The complete sequence of the *Cydia pomonella* granulovirus genome. J. Gen. Virol..

[29] Wang Y., Choi J.Y., Roh J.Y., Liu Q., Tao X.Y., Park J.B., Kim J.S., Je Y.H. (2011). Genomic sequence analysis of granulovirus isolated from the tobacco cutworm, *Spodoptera litura*. PLOS ONE.

[30] Wormleaton S., Kuzio J., Winstanley D. (2003). The complete sequence of the *Adoxophyes orana* granulovirus genome. Virology.

[31] Zhang B.-Q., Cheng R.-L., Wang X.-F., Zhang C.-X. (2012). The genome of *Pieris rapae* granulovirus. J. Virol..

[32] Zhang X., Liang Z., Yin X., Wang J., Shao X. (2014). Complete genome sequence of *Agrotis segetum* granulovirus shanghai strain. Arch. Virol..

[33] Greene G.L., Leppla N.C., Dickerson W.A. (1976). Velvetbean caterpillar (*Lepidoptera*, *noctuidae*) rearing procedure and artificial medium. J. Econ. Entomol..

[34] Hughes P.R., Wood H.A., Granados R.R., Federici B.B. (1986). *In vivo* and* in vitro* bioassay methods for baculoviruses. The Biology of Baculoviruses.

[35] Espinel-Correal C., Léry X., Villamizar L., Gómez J., Zeddam J.L., Cotes A.M., López-Ferber M. (2010). Genetic and biological analysis of colombian *Phthorimaea operculella* granulovirus isolated from tecia solanivora (lepidoptera: Gelechiidae). Appl. Environ. Microbiol..

[36] Caballero P., Zuidema D., Santiago-Alvarez C., Vlak J.M. (1992). Biochemical and biological characterization of four isolates of *Spodoptera exigua* nuclear polyhedrosis virus. Biocontrol Sci. Technol..

[37] Carver T., Berriman M., Tivey A., Patel C., Böhme U., Barrell B.G., Parkhill J., Rajandream M.-A. (2008). Artemis and act: Viewing, annotating and comparing sequences stored in a relational database. Bioinformatics.

[38] Fickett J.W. (1982). Recognition of protein coding regions in DNA sequences. Nucleic Acids Res..

[39] Altschul S.F., Gish W., Miller W., Myers E.W., Lipman D.J. (1990). Basic local alignment search tool. J. Mol. Biol..

[40] Larkin M., Blackshields G., Brown N., Chenna R., McGettigan P.A., McWilliam H., Valentin F., Wallace I.M., Wilm A., Lopez R. (2007). Clustal w and clustal x version 2.0. Bioinformatics.

[41] Thompson J., Higgins D., Gibson T. (1994). Clustal w: Improving the sensitivity of progressive multiple sequence alignment through sequence weighting, position-specific gap penalties and weight matrix choice. Nucleic Acids Res..

[42] Garavaglia M.J., Miele S.A.B., Iserte J.A., Belaich M.N., Ghiringhelli P.D. (2012). The *ac53*, *ac78*,* ac101*, and *ac103* genes are newly discovered core genes in the family *Baculoviridae*. J. Virol..

[43] National Center for Biotechnology Information. http://www.ncbi.nlm.nih.gov.

[44] Tamura K., Peterson D., Peterson N., Stecher G., Nei M., Kumar S. (2011). Mega5: Molecular evolutionary genetics analysis using maximum likelihood, evolutionary distance, and maximum parsimony methods. Mol. Biol. Evol..

[45] Tamura K., Stecher G., Peterson D., Filipski A., Kumar S. (2013). Mega6: Molecular evolutionary genetics analysis version 6.0. Mol. Biol. Evol..

[46] Gonnet G.H., Cohen M.A., Benner S.A. (1992). Exhaustive matching of the entire protein sequence database. Science.

[47] Zuker M., Mathews D.H., Turner D.H. (1999). Algorithms and thermodynamics for RNA secondary structure prediction: A practical guide. RNA Biochemistry and Biotechnology.

[48] Matzura O., Wennborg A. (1996). Rnadraw: An integrated program for RNA secondary structure calculation and analysis under 32-bit microsoft windows. Comput. Appl. Biosci..

[49] Thompson J.D., Gibson T.J., Plewniak F., Jeanmougin F., Higgins D.G. (1997). The clustal_x windows interface: Flexible strategies for multiple sequence alignment aided by quality analysis tools. Nucleic Acids Res..

[50] Crooks G.E., Hon G., Chandonia J.-M., Brenner S.E. (2004). Weblogo: A sequence logo generator. Genome Res..

[51] Lole K.S., Bollinger R.C., Paranjape R.S., Gadkari D., Kulkarni S.S., Novak N.G., Ingersoll R., Sheppard H.W., Ray S.C. (1999). Full-length human immunodeficiency virus type 1 genomes from subtype c-infected seroconverters in india, with evidence of intersubtype recombination. J. Virol..

[52] Salminen M.O., Koch C., Sanders-Buell E., Ehrenberg P.K., Michael N.L., Carr J.K., Burke D.S., McCutchan F.E. (1995). Recovery of virtually full-length HIV-1 provirus of diverse subtypes from primary virus cultures using the polymerase chain reaction. Virology.

[53] Cornette J.L., Cease K.B., Margalit H., Spouge J.L., Berzofsky J.A., DeLisi C. (1987). Hydrophobicity scales and computational techniques for detecting amphipathic structures in proteins. J. Mol. Biol..

[54] Eisenberg D., Weiss R.M., Terwilliger T.C. (1984). The hydrophobic moment detects periodicity in protein hydrophobicity. Proc. Natl. Acad. Sci. USA.

[55] Kyte J., Doolittle R.F. (1982). A simple method for displaying the hydropathic character of a protein. J. Mol. Biol..

[56] Petersen T.N., Brunak S., von Heijne G., Nielsen H. (2011). Signalp 4.0: Discriminating signal peptides from transmembrane regions. Nat. Methods.

[57] Söding J. (2005). Protein homology detection by hmm–hmm comparison. Bioinformatics.

[58] Wu S., Zhang Y. (2007). Lomets: A local meta-threading-server for protein structure prediction. Nucleic Acids Res..

[59] Roy A., Kucukural A., Zhang Y. (2010). I-tasser: A unified platform for automated protein structure and function prediction. Nat. Protoc..

[60] Xu D., Zhang Y. (2012). Ab initio protein structure assembly using continuous structure fragments and optimized knowledge- based force field. Proteins Struct. Funct. Bioinform..

[61] Friesen P.D., Miller L.K. (1997). Regulation of baculovirus early gene expression. The Baculoviruses.

[62] Lu A., Miller L.K., Krell P., Vlak J.M., Rohrmann G.F., Miller L.K. (1997). Baculovirus DNA replication. The Baculoviruses.

[63] Van Oers M.M., Vlak J.M. (2007). Baculovirus genomics. Curr. Drug Targets.

[64] Miele S.A.B., Garavaglia M.J., Belaich M.N., Ghiringhelli P.D. (2011). Baculovirus: Molecular insights on their diversity and conservation. Int. J. Evol. Biol..

[65] Herniou E.A., Jehle J.A. (2007). Baculovirus phylogeny and evolution. Curr. Drug Targets.

[66] Berretta M., Romanowski V. (2008). Baculovirus homologous regions (*hrs*): Pleiotropic functional cis elements in viral genomes and insect and mammalian cells. Curr. Top. Virol..

[67] Hilton S., Winstanley D. (2008). The origins of replication of granuloviruses. Arch. Virol..

[68] Wang Y., Choi J.Y., Roh J.Y., Woo S.D., Jin B.R., Je Y.H. (2008). Molecular and phylogenetic characterization of *Spodoptera litura* granulovirus. J. Microbiol..

[69] Liu X., Yin F., Zhu Z., Hou D., Wang J., Zhang L., Wang M., Wang H., Hu Z., Deng F. (2014). Genomic sequencing and analysis of *Sucra jujuba* nucleopolyhedrovirus. PLOS ONE.

[70] Harrison R.L. (2009). Structural divergence among genomes of closely related baculoviruses and its implications for baculovirus evolution. J. Invertebr. Pathol..

[71] Herniou E.A., Olszewski J.A., Cory J.S., O’Reilly D.R. (2003). The genome sequence and evolution of baculoviruses. Ann. Rev. Entomol..

[72] Maeda S., Kamita S.G., Kondo A. (1993). Host range expansion of *autographa californica* nuclear polyhedrosis virus (npv) following recombination of a 0.6-kilobase-pair DNA fragment originating from *Bombyx mori* npv. J. Virol..

[73] Xu Y.-P., Gu L.-Z., Lou Y.-H., Cheng R.-L., Xu H.-J., Wang W.-B., Zhang C.-X. (2012). A baculovirus isolated from wild silkworm encompasses the host ranges of *Bombyx mori* nucleopolyhedrosis virus and *Autographa californica* multiple nucleopolyhedrovirus in cultured cells. J. Gen. Virol..

[74] Jehle J.A., Fritsch E., Huber J., Backhaus H. (2003). Intra-specific and inter-specific recombination of tortricid-specific granuloviruses during co-infection in insect larvae. Arch. Virol..

[75] Croizier G., Croizier L., Quiot J.M., Lereclus D. (1988). Recombination of *Autographa californica* and *Rachiplusia ou* nuclear polyhedrosis viruses in *galleria mellonella* l. J. Gen. Virol..

[76] Meijer M., Karimi-Busheri F., Huang T.Y., Weinfeld M., Young D. (2002). DNA: Replication, repair, and recombination-pnk1, a DNA kinase/phosphatase required for normal response to DNA damage by g-radiation or camptothecin in *Schizosaccharomyces** pombe*. J. Biol. Chem..

