# Supplementary material for: The Complete Sequence of the First Spodoptera frugiperda Betabaculovirus Genome: A Natural Multiple Recombinant Virus
Source: Viruses. 2015 Jan 20;7(1):394–421. doi: 10.3390/v7010394 (PMC4306845; doi:10.3390/v7010394)
Supplement: Supplementary File 1 [file viruses-07-00394-s001.pdf]

Supplementary Material
The Complete Sequence of the First *Spodoptera frugiperda Betabaculovirus* Genome: A Natural Multiple Recombinant Virus

Paola E. Cuartas, Gloria P. Barrera, Mariano N. Belaich, Emiliano Barreto, Pablo D. Ghiringhelli and Laura F. Villamizar

Table S1. Potentially expressed ORFs in the genome of SfGV VG008.

ORF	Polarity (+/−)	Position		Gene Name	Prot. Size (aa)	Promoter Motifs			Orthologous Genes (% Identity/% Similatity)			
		Start	End			TATA Box	Early INR	Late INR	HearGV NC_010240	PsunGV NC_013772	SpliGV NC_009503	XecnGV NC_002331
001	+	1	747	*Granulin*	248		X	X	hear1 (96.4/100.0)	ORF1 (96.4/100.0)	gp001 (90.0/98.4)	ORF1 (96.4/100.0)
002	+	1384	2247	*pk-*	287		X (2)	X	hear3 (64.7/88.4)	ORF3 (68.6/94.1)	gp003 (62.4/85.2)	ORF3 (64.4/89.4)
003	−	1403	795	*ORF1629*	202		X (2)	X	hear2 (43.0/65.5)	ORF2 (43.4/66.4)	gp002 (38.0/69.8)	ORF2 (42.3/65.8)
004	−	3187	2288	*ORF004*	299	X	X (3)		---	ORF4 (31.7/61.1)	---	---
005	+	3284	3505	*p10*	73		X	X (2)	hear5 (61.9/82.1)	ORF5 (46.2/58.0)	gp004 (59.5/83.5)	ORF5 (63.1/82.1)
006	+	4073	4327	*ORF006*	84		X (2)		hear7 (54.7/83.7)	ORF7 (54.7/86.0)	---	ORF8 (55.8/83.7)
007	−	4083	3514	*ORF007*	189		X		hear6 (62.4/89.4)	ORF6 (60.8/88.9)	gp005 (42.1/75.8)	ORF7 (62.4/89.4)
008	−	5765	4320	*ie-1*	481		X (2)	X	hear8 (53.9/81.2)	ORF8 (53.4/80.5)	gp007 (35.7/68.7)	ORF9 (54.2/81.8)
009	+	5781	6353	*ORF009*	190		X	X	hear9 (56.7/83.6)	ORF9 (56.0/83.5)	gp008 (34.0/70.8)	ORF10 (55.7/83.6)
010	−	6670	6371	*chit-1*	99				hear10 (82.8/96.0)	ORF10 (83.8/97.0)	gp009 (65.7/90.9)	ORF11 (83.8/97.0)
011	−	6937	6680	*odv-e18*	85		X	X (2)	hear11 (76.7/88.4)	ORF11 (77.6/89.4)	gp010 (71.8/90.6)	ORF12 (77.9/88.4)

Table S1. *Cont.*

ORF	Polarity (+/−)	Position		Gene Name	Prot. Size (aa)	Promoter Motifs			Orthologous Genes (% Identity/% Similatity)			
		Start	End			TATA Box	Early INR	Late INR	HearGV NC_010240	PsunGV NC_013772	SpliGV NC_009503	XecnGV NC_002331
012	−	8302	6938	*49k*	454			X	hear12 (68.8/91.6)	ORF12 (70.3/92.7)	gp011 (47.2/79.5)	ORF13 (67.9/91.6)
013	−	8996	8319	*ORF013*	225				hear13 (60.7/85.2)	ORF13 (61.1/83.8)	gp012 (25.1/63.8)	ORF14 (61.6/85.2)
014	−	10077	9016	*odv-e56 /pif-5*	353		X (2)	X	hear14 (68.0/91.2)	ORF14 (68.8/92.1)	gp013 (53.3/80.7)	ORF15 (70.0/92.1)
015	+	10118	10330	*ORF015*	70	X	X		---	ORF15 (62.9/90.0)	---	---
016	−	10932	10342	*pep1*	196		X	X	hear16 (70.1/86.3)	ORF16 (69.8/86.9)	gp015 (51.6/73.1)	ORF17 (70.1/86.3)
017	−	11423	10968	*pep2*	151			X	hear17 (81.7/96.7)	ORF17 (82.4/96.7)	gp018 (53.0/81.5)	ORF18 (82.4/96.7)
018	−	12601	11444	*pep/p10*	385		X	X	hear18 (76.5/93.3)	ORF18 (77.5/93.3)	gp019 (57.6/83.2)	ORF19 (76.3/93.3)
019	−	13542	12676	*ORF019*	288				hear22 (32.4/64.7)	ORF22 (31.8/59.9)	---	ORF23 (32.4/62.8)
020	−	14011	13679	*ORF020*	110		X		hear23 (50.0/73.4)	ORF23 (48.2/81.3)	---	ORF24 (50.0/73.4)
021	+	14888	16183	*ORF021*	431		X		hear24 (37.7/67.4)	ORF24 (43.0/70.4)	gp022 (24.1/61.8)	ORF25 (38.8/69.9)
022	+	16620	17507	*ORF022*	295	X	X (4)		hear25 (21.2/53.2)	ORF25 (25.3/58.9)	---	ORF26 (22.0/53.2)
023	+	17485	19323	*efp*	612		X		hear26 (52.1/80.8)	ORF26 (52.8/81.6)	gp024 (29.1/67.5)	ORF27 (52.6/81.8)
024	+	19351	20169	*ORF024*	272				---	---	---	---

Table S1. *Cont.*

ORF	Polarity (+/−)	Position		Gene Name	Prot. Size (aa)	Promoter Motifs			Orthologous Genes (% Identity/% Similatity)			
						TATA Box	Early INR	Late INR	HearGV	PsunGV	SpliGV	XecnGV
		Start	End						NC_010240	NC_013772	NC_009503	NC_002331
025	−	20852	20166	*ORF025*	228			X	hear28 (55.2/83.8)	ORF28 (55.7/86.1)	gp026 (38.5/68.4)	ORF29 (42.1/66.2)
026	−	21450	20869	*ORF026*	193		X	X	hear29 (69.9/91.2)	ORF29 (75.6/94.3)	---	ORF30 (44.0/59.6)
027	+	21468	22046	*pif-3*	192		X (2)	X	hear30 (60.5/87.7)	ORF30 (62.1/89.2)	gp027 (38.1/67.0)	ORF32 (59.5/87.7)
028	+	22053	22355	*ORF028*	100		X	X	---	---	---	---
029	+	22359	22694	*ORF029*	111		X	X	hear32 (80.5/93.8)	ORF32 (80.5/93.8)	gp031 (44.6/72.7)	ORF34 (80.5/93.8)
030	+	22697	23293	*lef-2*	198		X		hear33 (56.3/81.9)	ORF33 (56.2/83.1)	gp032 (40.6/68.3)	ORF35 (56.3/81.9)
031	+	23226	23549	*ORF031*	107		X (2)		---	---	---	---
032	-	23896	23621	*CIDE_N*	91			X (2)	hear36 (47.7/67.6)	ORF36 (48.6/71.0)	---	ORF38 (45.9/68.5)
033	−	24466	24056	*ORF033*	136		X		hear37 (46.0/75.3)	ORF37 (45.4/76.3)	---	ORF39 (46.0/76.0)
034	−	26163	24544	*mp-nase*	539				hear38 (48.3/72.8)	ORF38 (47.8/73.6)	gp037 (33.6/65.4)	ORF40 (46.8/69.4)
035	−	27337	26246	*ORF035*	363		X	X (2)	hear39 (24.6/63.4)	ORF40 (20.5/52.2)	---	ORF42 (23.9/61.7)
036	+	27418	28260	*p13*	280		X (2)	X (2)	hear40 (71.1/90.7)	ORF41 (70.7/91.8)	gp038 (50.5/76.7)	ORF43 (70.7/91.1)
037	+	29750	30892	*pif-2*	380			X	hear42 (75.0/88.4)	ORF43 (75.3/88.7)	gp039 (57.4/81.8)	ORF45 (74.7/88.1)
038	+	31146	34580	*ORF038*	1144		X	X	hear44 (32.3/64.2)	ORF45 (34.8/66.4)	gp041 (15.4/45.6)	ORF46,ORF47 (28.6/58.9) ‡

Table S1. *Cont.*

ORF	Polarity (+/−)	Position		Gene Name	Prot. Size (aa)	Promoter Motifs			Orthologous Genes (% Identity/% Similatity)			
						TATA Box	Early INR	Late INR	HearGV NC_010240	PsunGV NC_013772	SpliGV NC_009503	XecnGV NC_002331
		Start	End									
039	−	35256	34573	*ORF039*	227			X	hear45 (69.5/82.2)	ORF47 (69.0/81.2)	gp042 (78.4/90.7)	ORF50 (66.4/77.4)
040	+	35304	35465	*ORF040*	53		X		---	---	---	---
041	+	35645	36889	*odv-ec43*	414				hear48 (59.6/75.2)	ORF51 (61.8/76.7)	gp046 (44.8/68.7)	ORF53 (59.6/75.0)
042	−	35736	35488	*v-ubi*	82			X (2)	hear47 (90.2/93.9)	ORF50 (87.8/93.9)	gp045 (76.3/84.9)	ORF52 (90.2/93.9)
043	+	36906	37211	*ORF043*	101		X (3)	X	hear49 (62.5/79.5)	ORF52 (62.5/79.5)	---	ORF54 (61.6/78.6)
044	−	38119	37244	*39k*	291		X	X	hear50 (65.2/87.3)	ORF53 (66.0/87.5)	gp048 (54.9/77.3)	ORF55 (65.4/87.0)
045	−	38384	38109	*lef-11*	91				hear51 (69.6/84.3)	ORF54 (73.7/91.6)	gp049 (42.9/78.6)	ORF56 (69.6/85.3)
046	+	38609	39562	*ORF046*	317	X	X (2)		---	---	---	---
047	+	40138	40929	*ORF047*	263	X (2)	X (2)	X	hear52 (27.5/61.2)	ORF55 (29.1/61.1)	---	ORF57 (27.4/63.3)
048	−	41546	41091	*dUTPase*	151		X (3)	X	---	---	gp051 (72.2/90.1)	---
049	+	41638	42501	*ORF049*	287	X (2)	X (2)		---	---	---	---
050	−	42945	42517	*sod*	142		X	X	hear63 (64.9/84.4)	ORF64 (63.4/81.7)	---	ORF68 (66.7/85.0)
051	+	43044	44444	*DNA photolyase*	466	X (2)			---	---	gp050 (76.9/94.0)	---
052	−	45237	44479	*broa*	252	X	X (2)		hear133 (15.7/37.7)	ORF66, ORF135 (17.1/35.7)	gp114 (36.5/71.9)	ORF130 (29.2/63.5)

Table S1. *Cont.*

ORF	Polarity (+/−)	Position		Gene Name	Prot. Size (aa)	Promoter Motifs			Orthologous Genes (% Identity/% Similatity)			
		Start	End			TATA Box	Early INR	Late INR	HearGV NC_010240	PsunGV NC_013772	SpliGV NC_009503	XecnGV NC_002331
053	-	46890	45322	*brob*	522		X (4)		hear133, hear54 (39.4/70.3)	ORF66, ORF158, ORF137 (49.3/72.3)	gp053, gp115, gp111 (35.0/54.5)	ORF131, ORF60 (38.2/67.3)
054	−	47924	47355	*ORF054*	189			X	---	---	---	---
055	+	48047	48601	*ORF055*	184		X (2)	X	---	---	---	---
056	+	48732	50000	*ORF056*	422		X	X	hear67 (38.4/62.5)	ORF72 (45.3/71.4)	---	ORF73 (48.2/76.4)
057	−	52230	50077	*broc*	717		X (4)		---	---	---	---
058	+	52398	52679	*endonuclease*	93	X			hear69 (58.6/76.8)	ORF74 (56.6/77.8)	---	ORF75 (57.6/77.8)
059	−	53537	53124	*ORF059*	137		X (2)		---	---	---	---
060	+	55418	56221	*brod*	267	X	X	X	hear71 (48.8/77.3)	ORF76 (48.6/75.0)	---	ORF76 (49.5/76.3)
061	+	56258	58372	*p74/pif-0*	704		X	X	hear72 (68.5/91.0)	ORF77 (67.5/90.7)	gp056 (9.1/17.3) §	ORF77 (68.5/90.9)
062	−	58607	58326	*ORF062*	93		X	X	hear73 (55.8/77.9)	ORF78 (48.6/73.0)	---	---
063	+	58679	59863	*p47*	394	X	X		hear74 (75.4/93.7)	ORF79 (75.4/93.2)	gp058 (60.8/79.9)	ORF78 (75.4/93.7)
064	+	59990	60673	*nudixPPi*	227		X	X	hear77 (91.6/98.2)	ORF82 (92.5/98.2)	gp059 (75.1/92.6)	ORF79 (92.1/98.7)
065	+	60750	61280	*p24*	176			X (2)	hear78 (60.3/83.1)	ORF83 (66.1/89.3)	gp060 (52.3/81.3)	ORF80 (60.3/83.1)
066	−	61825	61298	*38.7k*	175		X (2)		hear79 (47.0/74.1)	ORF84 (44.8/73.4)	---	ORF81 (45.4/74.6)

Table S1. *Cont.*

ORF	Polarity (+/−)	Position		Gene Name	Prot. Size (aa)	Promoter Motifs			Orthologous Genes (% Identity/% Similatity)			
		Start	End			TATA Box	Early INR	Late INR	HearGV NC_010240	PsunGV NC_013772	SpliGV NC_009503	XecnGV NC_002331
067	−	62543	61827	*lef-1*	238		X (3)		hear80 (72.0/92.1)	ORF85 (74.1/91.6)	gp062 (46.6/70.8)	ORF82 (71.5/91.2)
068	+	62608	63129	*p10*	173		X	X (2)	hear81 (52.9/82.0)	ORF86 (47.2/83.4)	---	ORF83 (52.4/81.5)
069	+	63153	64778	*pif-1*	541		X	X	hear82 (59.8/85.5)	ORF87 (59.1/84.8)	gp064 (42.2/76.2)	ORF84 (59.4/85.0)
070	−	65502	64816	*fgf-1*	228		X (2)		hear83 (42.3/75.6)	ORF88 (42.7/76.9)	go066 (29.3/65.5)	ORF85 (42.3/76.9)
071	+	65573	66061	*chit-2a*	162			X	hear85 (26.2/63.1)	ORF90 (27.8/60.9)	---	ORF87 (25.6/61.9)
072	+	66129	66608	*chit-2b*	159		X	X (2)	hear85 (35.9/62.9)	ORF90 (38.2/65.3)	---	ORF87 (31.8/61.2)
073	−	66904	66605	*lef-6*	99				hear86 (60.4/88.1)	ORF91 (56.9/87.3)	---	ORF88 (61.4/88.1)
074	−	67804	66959	*dbp*	281	X	X (2)		hear87 (39.0/77.7)	ORF92 (37.9/77.3)	gp070 (23.4/64.9)	ORF89 (39.7/79.1)
075	+	68680	69798	*p48/p45*	372		X	X	hear90 (82.5/96.2)	ORF95 (80.4/96.2)	gp073 (64.4/92.2)	ORF91 (83.3/96.2)
076	−	68681	68010	*ORF076*	223		X	X	hear89 (33.6/64.4)	ORF94 (37.1/67.3)	---	ORF90 (34.0/64.4)
077	+	69806	70141	*p12*	111		X	X	hear91 (62.3/83.6)	ORF96 (60.7/79.5)	gp074 (52.2/81.7)	ORF92 (62.8/84.3)
078	+	70193	71311	*odv-ec42/p40*	372			X	hear92 (78.0/93.8)	ORF97 (77.2/93.8)	gp075 (60.9/87.4)	ORF93 (76.5/92.5)
079	+	71334	71510	*p6.9*	58			X (2)	hear93 (80.0/90.0)	ORF98 (83.3/90.0)	---	ORF94 (81.7/90.0)

Table S1. *Cont.*

ORF	Polarity (+/−)	Position		Gene Name	Prot. Size (aa)	Promoter Motifs			Orthologous Genes (% Identity/% Similatity)			
		Start	End			TATA Box	Early INR	Late INR	HearGV NC_010240	PsunGV NC_013772	SpliGV NC_009503	XecnGV NC_002331
080	+	72254	73168	*38k*	304		X	X	hear95 (65.4/89.5)	ORF100 (64.1/89.2)	gp077 (55.1/79.7)	ORF96 (65.7/88.9)
081	−	72331	71549	*lef-5*	260		X (3)		hear94 (72.4/87.5)	ORF99 (71.5/87.8)	gp076 (58.0/80.2)	ORF95 (62.1/76.1)
082	+	73642	77136	*DNAhel-1*	1164		X (3)		hear97 (74.4/92.0)	ORF102 (75.1/92.6)	gp080 (64.9/87.6)	ORF98 (74.3/92.0)
083	−	73643	73170	*pif-4*	157			X	hear96 (77.7/94.9)	ORF101 (77.7/94.9)	gp079 (52.5/85.6)	ORF97 (77.1/94.9)
084	+	77227	78027	*lef-7*	266	X	X		hear132 (24.3/49.2)	---	---	ORF129 (24.3/49.2)
085	−	78696	78037	*odv-e25*	219		X (2)	X (2)	hear98 (82.7/98.2)	ORF103 (80.8/96.8)	gp081 (67.3/92.3)	ORF99 (81.4/97.7)
086	+	79150	80061	*p33*	303		X		hear100 (59.2/77.3)	ORF105 (60.7/77.9)	gp083 (42.9/71.0)	ORF101 (59.7/77.9)
087	−	79229	78753	*p18*	158			X	hear99 (67.1/94.9)	ORF104 (66.5/94.9)	gp082 (50.6/83.1)	ORF100 (50.0/72.8)
088	−	80286	80047	*chaB*	79		X (4)	X	hear103 (69.0/83.9)	ORF106 (68.5/83.1)	---	ORF102 (69.0/82.8)
089	−	80780	80310	*ORF089*	156		X		hear131 (31.4/67.9)	ORF132 (31.7/67.7)	---	ORF128 (33.3/69.8)
090	−	82848	81235	*broe*	537	X	X	X	hear54, hear133 (37.8/70.4)	ORF137 (45.0/73.5)	gp111, gp115 (45.1/70.9)	ORF60, ORF131 (44.0/71.5)
091	−	84465	83140	*lef-4*	441			X	hear112 (65.6/87.2)	ORF114 (65.5/88.4)	gp086 (51.1/81.8)	ORF110 (66.1/87.1)

Table S1. *Cont.*

ORF	Polarity (+/−)	Position		Gene Name	Prot. Size (aa)	Promoter Motifs			Orthologous Genes (% Identity/% Similatity)			
		Start	End			TATA Box	Early INR	Late INR	HearGV NC_010240	PsunGV NC_013772	SpliGV NC_009503	XecnGV NC_002331
092	+	84521	85495	*vp39*	324		X (2)	X (3)	hear113 (68.7/88.1)	ORF115 (71.3/87.5)	gp087 (56.7/80.7)	ORF111 (69.3/87.5)
093	+	85568	86440	*odv-ec27 /odv-e27*	290		X (2)		hear114 (79.7/97.2)	ORF116 (80.3/97.6)	gp088 (64.8/91.0)	ORF112 (79.7/97.6)
094	−	87775	86663	*brof*	370		X	X	hear116 (52.7/82.7)	ORF117 (48.4/76.2)	gp089 (33.0/69.1)	ORF113 (52.3/82.5)
095	−	89182	88118	*brog*	354		X (2)	X (2)	hear117 (64.5/86.7)	ORF118 (56.7/78.0)	---	ORF114 (57.9/77.8)
096	+	89305	90462	*ORF096*	385				hear118 (51.0/77.3)	ORF119 (49.6/74.7)	---	ORF115 (49.9/75.3)
097	+	90521	90895	*ORF097*	124			X (2)	hear119 (70.9/89.8)	ORF120 (69.3/90.6)	---	ORF116 (70.1/89.8)
098	−	91473	90877	*ORF098*	198				---	---	---	---
099	−	92250	91654	*ORF099*	198			X	---	---	---	---
100	+	94401	94940	*tlp20*	179	X	X		hear122 (57.8/77.2)	ORF123 (57.2/78.9)	gp093 (30.1/66.1)	ORF119 (56.0/75.8)
101	-	94432	92276	*vp91/p95*	718		X (2)	X	hear121 (55.8/81.9)	ORF122 (53.8/80.8)	gp092 (32.7/59.1)	ORF118 (55.1/81.5)
102	+	94991	95566	*ORF102*	191		X	X	hear123 (77.0/95.3)	ORF124 (76.4/95.3)	gp094 (62.7/91.7)	ORF120 (77.0/95.3)
103	+	95595	96479	*gp41*	294		X	X	hear124 (74.1/91.5)	ORF125 (72.8/90.8)	gp095 (66.7/89.8)	ORF121 (74.1/91.5)
104	+	96553	96864	*ORF104*	103		X (2)	X (2)	hear125 (56.7/83.7)	ORF126 (56.1/80.4)	---	ORF122 (55.8/83.7)
105	+	96836	97942	*vlf-1*	368		X	X	hear126 (77.7/93.6)	ORF127 (77.5/94.6)	gp097 (64.8/90.7)	ORF123 (78.1/93.6)

Table S1. *Cont.*

ORF	Polarity (+/−)	Position		Gene Name	Prot. Size (aa)	Promoter Motifs			Orthologous Genes (% Identity/% Similatity)			
		Start	End			TATA Box	Early INR	Late INR	HearGV NC_013772	PsunGV NC_009503	SpliGV NC_002331	XecnGV NC_013772
106	+	98509	98766	*ORF106*	85		X (2)	X	hear128 (89.4/98.8)	ORF129 (88.2/97.6)	gp099 (81.2/97.6)	ORF125 (89.4/98.8)
107	−	98603	97932	*ORF107*	223		X (2)		hear127 (47.0/67.0)	ORF128 (45.2/64.8)	gp098 (29.8/55.7)	ORF124 (46.1/67.0)
108	+	98806	99243	*ORF108*	145		X	X	hear129 (69.7/92.4)	ORF130 (71.7/92.4)	gp100 (43.2/84.5)	ORF126 (69.7/92.4)
109	−	102475	99278	*DNApol*	1065		X (2)		hear134 (70.3/88.7)	ORF138 (69.8/88.3)	gp101 (59.7/84.1)	ORF132 (70.5/88.8)
110	+	102474	104387	*desmoplakin*	637		X		hear135 (49.1/77.5)	ORF139 (49.1/77.6)	gp102 (24.8/63.5)	ORF133 (49.3/77.4)
111	+	105432	105851	*pif-6*	139				hear137 (67.6/92.8)	ORF141 (70.5/93.5)	gp104 (44.3/80.7)	ORF135 (54.7/79.1)
112	−	105466	104414	*lef-3*	350		X (2)		hear136 (44.7/79.8)	ORF140 (44.9/78.1)	gp103 (38.4/70.7)	ORF134 (44.4/79.8)
113	+	105888	106388	*ORF113*	166			X (2)	hear138 (37.6/67.6)	ORF142 (39.2/70.2)	---	ORF136 (38.7/67.6)
114	+	106449	107318	*iap-3*	289		X		hear139 (53.4/77.6)	ORF143 (52.3/77.9)	gp106 (33.4/65.2)	ORF137 (53.2/78.6)
115	+	107210	108841	*lef-9*	543				hear140 (72.5/84.2)	ORF145 (72.6/85.0)	gp107 (63.0/82.8)	ORF139 (72.7/84.2)
116	+	108825	109337	*fp*	170		X (2)		hear141 (72.9/82.4)	ORF146 (34.1/38.2)	gp109 (44.0/66.9)	ORF140 (71.2/81.8)
117	−	110912	109339	*DNAligase*	534		X (2)	X	hear142 (67.8/89.6)	ORF148 (66.4/87.1)	gp110 (47.0/73.1)	ORF141 (67.8/89.3)
118	+	111094	111294	*ORF118*	66	X	X		---	---	---	---

Table S1. *Cont.*

ORF	Polarity (+/−)	Position		Gene Name	Prot. Size (aa)	Promoter Motifs			Orthologous Genes (% Identity/% Similatity)			
						TATA	Early	Late	HearGV	PsunGV	SpliGV	XecnGV
		Start	End			Box	INR	INR	NC_013772	NC_009503	NC_002331	NC_013772
119	+	111355	111555	*ORF119*	66		X	X	hear144 (68.2/86.4)	ORF150 (68.2/86.4)	---	ORF143 (68.2/86.4)
120	−	112754	111591	*fgf-2*	387				hear145 (49.4/79.0)	ORF151 (51.2/81.9)	gp116 (39.0/72.5)	ORF144 (49.1/78.5)
121	+	112880	114097	*alk-exo*	405		X	X	hear146 (52.7/77.7)	ORF152 (54.8/76.7)	gp117 (44.8/77.0)	ORF145 (51.9/77.7)
122	+	114140	115552	*DNAhel-2*	470	X	X (3)		hear147 (64.5/86.4)	ORF153 (63.8/86.4)	gp118 (50.3/79.9)	ORF146 (63.7/86.6)
123	+	115653	116654	*ORF123*	333	X	X (2)		hear148 (56.0/80.8)	ORF154 (55.6/82.1)	gp120 (36.0/70.5)	ORF147 (47.3/73.5)
124	−	119253	116674	*lef-8*	859			X (3)	hear149 (82.2/93.3)	ORF155 (81.3/93.8)	gp121 (68.8/88.1)	ORF148 (82.1/93.1)
125	−	120425	119409	*ORF125*	338				---	---	---	---
126	−	122496	120472	*odv-e66*	674		X	X	hear150 (69.8/89.8)	ORF156 (70.0/90.1)	gp125 (18.0/52.29)	ORF149 (69.8/89.7)
127	−	125378	122775	*enhancin-1*	867		X (2)	X	hear152 (44.4/76.7)	ORF159, ORF157 (28.5/61.2)	---	ORF152 (44.5/77.1)
128	+	125607	127145	*ORF128*	512			X	hear160 (54.8/80.9)	ORF164, ORF021 (51.3/79.1)	---	ORF161, ORF22 (55.2/80.9)
129	+	127194	128198	*ORF129*	334	X	X		---	---	---	---
130	−	128882	128220	*ORF130*	220		X		hear161 (35.0/65.0)	ORF165 (37.3/68.2)	---	ORF162 (35.0/65.0)
131	+	129167	129652	*ORF131*	161		X (2)		hear163 (47.8/67.7)	ORF169 (47.8/67.1)	---	ORF165 (47.2/67.7)

Table S1. *Cont.*

ORF	Polarity (+/−)	Position		Gene Name	Prot. Size (aa)	Promoter Motifs			Orthologous Genes (% Identity/% Similatity)			
		Start	End			TATA Box	Early INR	Late INR	HearGV NC_013772	PsunGV NC_009503	SpliGV NC_002331	XecnGV NC_013772
132	−	132258	129685	*enhancin-2*	857		X (3)	X (3)	hear164 (40.5/75.5)	ORF170 (41.0/75.7)	---	ORF166 (40.6/74.7)
133	+	132403	132852	*ORF133*	149	X	X (2)	X	---	---	---	---
134	+	132909	133397	*chit-2c*	162		X	X	---	---	---	---
135	+	133671	134261	*U-box /RING-like*	196		X (2)		hear169 (42.6/61.9)	ORF173 (43.9/63.8)	gp127 (35.9/65.2)	ORF171 (43.1/61.9)
136	−	135495	134242	*ORF136*	417		X		hear170 (50.9/78.2)	ORF174 (54.0/78.2)	gp128 (23.7/55.2)	ORF172 (50.7/78.6)
137	+	135683	135901	*lef-10*	72			X	hear172 (72.2/86.1)	ORF176 (73.6/88.9)	---	ORF174 (72.2/87.5)
138	−	135705	135502	*ORF138*	67		X	X	hear171 (68.7/95.5)	ORF175 (76.1/94.0)	---	ORF173 (68.7/95.5)
139	+	135774	136748	*vp1054*	324		X	X	hear173 (74.2/90.8)	ORF177 (75.4/91.7)	gp130 (48.9/80.4)	ORF175 (74.5/90.5)
140	+	136834	137019	*ORF140*	61		X (2)	X	---	ORF178 (67.2/95.1)	---	---
141	+	137012	137422	*ORF141*	136				---	---	---	---
142	+	137456	138361	*fgf-3*	301		X (3)		hear176 (41.3/74.2)	ORF180 (44.1/83.6)	gp133 (39.6/73.)	ORF178 (43.3/78.6)
143a	+	138378	139067	*ORF143a*	229		X		---	---	---	---
143b	+	139070	139690	*ORF143b*	206	X	X		hear177 (29.3/66.8)	ORF181 (27.6/63.3)	---	ORF179 (27.4/62.0)
144	+	139694	140590	*me53*	298				hear178 (60.4/81.7)	ORF182 (65.8/89.3)	gp134 (40.3/73.6)	ORF180 (61.0/81.7)
145	+	140592	140891	*ORF145*	99		X	X	hear179 (56.9/84.4)	ORF183 (59.8/84.1)	---	ORF181 (56.4/83.6)

‡ ORFs 47 and 48 of XcGV were concatenated. § ORF p74 in SpliGV is truncated. Rows shaded in green indicate unique ORFs of SfGV VG008. Rows shaded in blue indicate ORFs only present in SfGV VG008 and PsunGV.

Table S2. *Baculoviridae* core genes in *Betabaculovirus* species.

Sfgvorf	Gene Name	Function	AdorGV ORF	AgseGV ORF	ChocGV ORF	ClanGV ORF	CalGV ORF	CrleGV ORF	CpGV ORF	EpapGV ORF	HearGV ORF	PhopGV ORF	PsunGV ORF	PiraGV ORF	PxGV ORF	SpliGV ORF	XecnGV ORF
30	*lef-2*	DNA replication/ primase-associated factor	32	35	29	30	29	38	41	41	33	37	33	33	32	32	35
67	*lef-1*	DNA primase	62	64	55	62	60	66	74	68	80	66	85	60	55	62	82
37	*pif-2*	Required for per os infection	39	43	35	24	23	45	48	47	42	44	43	40	37	39	45
63	*p47*	RNA polymerase subunit	58	60	50	57	55	61	68	63	74	61	79	56	51	58	78
124	*lef-8*	RNA polymerase subunit	110	118	107	111	111	119	131	121	149	121	155	110	109	121	148
135	*ac53*	Likely involved in nucleocapsid assembly/Ubox/ RING-like domain	111	122	109	114	115	121	134	124	169	122	173	113	112	127	171
139	*vp1054*	Nucleocapsid protein	115	127	113	118	119	125	138	128	173	126	177	116	115	130	175
115	*lef-9*	RNA polymerase subunit	100	107	96	101	101	107	117	112	140	109	145	99	99	107	139

Table S2. *Cont.*

Sfgvorf	Gene Name	Function	AdorGV ORF	AgseGV ORF	ChocGV ORF	ClanGV ORF	CalGV ORF	CrleGV ORF	CpGV ORF	EpapGV ORF	HearGV ORF	PhopGV ORF	PsunGV ORF	PiraGV ORF	PxGV ORF	SpliGV ORF	XecnGV ORF
109	*dnapol*	DNA replication	94	101	90	96	95	101	111	106	134	103	138	93	93	101	132
110	*desmoplakin*	Present in nucleocapsid	95	102	91	97	96	102	112	107	135	104	139	94	94	102	133
111	*ac68/pif-6*	Required for per os infection	97	104	93	99	98	104	114	109	137	106	141	96	96	104	135
105	*Vlf1*	Involved in expression of the p10 and polh genes	91	97	86	92	91	97	106	101	126	99	127	90	89	97	123
104	*ac78*	Unknown function/ transmembrane domain	90	96	85	90	37	96	105	43	125	98	126	89	88	96	122
103	*gp41*	Tegument protein	88	95	83	89	89	95	104	99	124	97	125	88	87	95	121
102	*ac81*	Unknown function	87	94	82	88	43	94	103	98	123	96	124	87	86	94	120
101	*p95*	Viral capsid-associated protein	85	91	80	86	87	92	101	96	121	94	122	85	84	92	118
92	*vp39*	Major capsid protein	81	86	76	83	81	87	96	92	113	88	115	81	79	87	111

Table S2. *Cont.*

Sfgvorf	Gene Name	Function	AdorGV ORF	AgseGV ORF	ChocGV ORF	ClanGV ORF	CalGV ORF	CrleGV ORF	CpGV ORF	EpapGV ORF	HearGV ORF	PhopGV ORF	PsunGV ORF	PiraGV ORF	PxGV ORF	SpliGV ORF	XecnGV ORF
91	*lef-4*	RNA polymerase subunit/capping enzyme	80	85	75	82	80	86	95	91	112	87	114	80	78	86	110
86	*p33*	Sulfhydryl oxidase	79	83	74	80	78	84	93	88	100	85	105	78	76	83	101
87	*p18*	Egress of nucleocapsids	78	82	73	77	79	83	92	87	99	84	104	77	75	82	100
85	*odv-e25*	ODV envelope protein	77	81	72	78	76	82	91	86	98	83	103	76	74	81	99
82	*helicase*	Unwinding DNA	76	79	71	77	75	81	90	85	97	82	102	75	72	80	98
83	*ac96/pif-4*	Required for per os infection	75	78	70	74	76	80	89	84	96	81	101	74	71	79	97
80	*38k*	Required for nucleocapsid assembly	74	77	69	75	73	79	88	83	95	80	100	73	70	77	96
81	*lef-5*	Transcription initiation factor	73	76	68	74	72	78	87	82	94	79	99	72	69	76	95
79	*p6.9*	Nucleocapsid protein	72	75	67	73	73	77	86	81	93	78	98	71	67	75b*	94
78	*p40*	Subunit of protein complex	71	74	66	72	70	76	85	80	92	77	97	70	66	75	93
75	*p48*	BV production and ODV envelopment	69	72	64	70	68	74	83	78	90	75	95	68	63	73	91

Table S2. *Cont.*

Sfgvorf	Gene Name	Function	AdorGV ORF	AgseGV ORF	ChocGV ORF	ClanGV ORF	CalGV ORF	CrleGV ORF	CpGV ORF	EpapGV ORF	HearGV ORF	PhopGV ORF	PsunGV ORF	PiraGV ORF	PxGV ORF	SpliGV ORF	XecnGV ORF
41	*odv-ec43*	Associated with ODV	46	48	40	46	46	53	55	53	48	50	51	46	43	46	53
27	*pif-3*	Required for per os infection	26	29	26	34	33	34	35	38	30	31	30	30	29	27	32
69	*pif-1*	Mediates binding of ODV to midgut	63	65	56	63	61	67	75	69	82	67	87	61	7	64	84
121	*alk exo*	Involved in DNA recombination and replication	107	115	104	109	109	115	125	119	146	114	152	107	106	117	145
61	*p74/pif-0*	Mediates binding of ODV to midgut	53	56	46	51	49	58	60	59	72	55	77	51	49	56	77
12	*49k*	Required for BV production	11	12	13	14	14	14	15	28	12	13	12	15	14	11	13
11	*odv-e18*	ODV envelope protein	10	11	12	13	13	13	14	29	11	12	11	14	13	10	12
93	*odv-e27*	ODV envelope protein	82	87	77	84	82	88	97	93	114	89	116	82	80	88	112
14	*odv-e56/ pif-5*	ODV envelope protein	12	15	14	15	15	17	18	27	14	16	14	16	16	13	15

* p6.9 was not annotated in the GenBank file.

Table S3. Orthologous genes in betabaculoviruses from Noctuide insects.

**SfGV VG008**				**Orthologous Genes**												
				**HearGV**			**PsunGV**			**SpliGV**			**XecnGV**			
**ORF**	**Gene Name**	**Polar. (+/−)**	**Prot. Size (aa)**	**Polar. (+/−)**	**Prot. Size (aa)**	**NC_010240**	**Polar. (+/−)**	**Prot. Size (aa)**	**NC_013772**	**Polar. (+/−)**	**Prot. Size (aa)**	**NC_009503**	**Polar. (+/-)**	**Prot. Size (aa)**	**NC_002331**
001	*Granulin*	+	248	+	248	hear1	+	248	ORF1	+	249	gp001	+	248	ORF1
002	*pk-1*	+	287	+	299	hear3	+	284	ORF3	+	282	gp003	+	302	ORF3
003	*ORF1629*	−	202	−	233	hear2	−	240	ORF2	−	189	gp002	−	231	ORF2
004	*ORF004*	−	299			---	−	291	ORF4			---			---
005	*p10*	+	73	+	84	hear5	+	119	ORF5	+	78	gp004	+	84	ORF5
006	*ORF006*	+	84	+	86	hear7	+	86	ORF7			---	+	86	ORF8
007	*ORF007*	−	189	−	187	hear6	−	187	ORF6	−	189	gp005	−	187	ORF7
008	*ie-1*	−	481	−	485	hear8	−	474	ORF8	−	432	gp007	−	484	ORF9
009	*ORF009*	+	190	+	196	hear9	+	195	ORF9	+	205	gp008	+	196	ORF10
010	*chit-1*	−	99	−	99	hear10	−	99	ORF10	−	99	gp009	−	99	ORF11
011	*odv-e18*	−	85	−	83	hear11	−	83	ORF11	−	80	gp010	−	83	ORF12
012	*49k*	−	454	−	453	hear12	−	452	ORF12	−	456	gp011	−	453	ORF13
013	*ORF013*	−	225	−	229	hear13	−	229	ORF13	−	227	gp012	−	229	ORF14
014	*odv-e56/ pif-5*	−	353	−	353	hear14	−	353	ORF14	−	365	gp013	−	353	ORF15
015	*ORF015*	+	70			---	+	68	ORF15			---			---
016	*pep1*	−	196	−	187	hear16	−	190	ORF16	−	215	gp015	−	187	ORF17
017	*pep2*	−	151	−	153	hear17	−	153	ORF17	−	144	gp018	−	153	ORF18
018	*pep/p10*	−	385	−	386	hear18	−	387	ORF18	−	370	gp019	−	386	ORF19
019	*ORF019*	−	288	−	341	hear22	−	357	ORF22			---	−	350	ORF23
020	*ORF020*	−	110	−	124	hear23	−	112	ORF23			---	−	124	ORF24

Table S3. *Cont.*

SfGV VG008				Orthologous Genes											
				HearGV			PsunGV			SpliGV			XecnGV		
**ORF**	**Gene Name**	**Polar. (+/−)**	**Prot. Size (aa)**	**Polar. (+/−)**	**Prot. Size (aa)**	**NC_010240**	**Polar. (+/−)**	**Prot. Size (aa)**	**NC_013772**	**Polar. (+/−)**	**Prot. Size (aa)**	**NC_009503**	**Polar. (+/-)**	**Prot. Size (aa)**	**NC_002331**
021	*ORF021*	+	431	+	469	hear24	+	437	ORF24	+	410	gp022	+	449	ORF25
022	*ORF022*	+	295	+	370	hear25	+	328	ORF25			---	+	370	ORF26
023	*efp*	+	612	+	581	hear26	+	581	ORF26	+	585	gp024	+	599	ORF27
024	*ORF024*	+	272			---			---			---			---
025	*ORF025*	−	228	−	240	hear28	−	236	ORF28	−	229	gp026	−	170	ORF29
026	*ORF026*	−	193	−	192	hear29	−	192	ORF29			---	−	128	ORF30
027	*pif-3*	+	192	+	195	hear30	+	194	ORF30	+	215	gp027	+	195	ORF32
028	*ORF028*	+	100			---			---			---			---
029	*ORF029*	+	111	+	113	hear32	+	113	ORF32	+	116	gp031	+	113	ORF34
030	*lef-2*	+	198	+	189	hear33	+	194	ORF33	+	174	gp032	+	189	ORF35
031	*ORF031*	+	107			---			---			---			---
032	*CIDE_N*	−	91	−	111	hear36	−	106	ORF36			---	−	111	ORF38
033	*ORF033*	−	136	−	149	hear37	−	152	ORF37			---	−	149	ORF39
034	*mp-nase*	−	539	−	596	hear38	−	593	ORF38	−	464	gp037	−	469	ORF40
035	*ORF035*	−	363	−	381	hear39	−	323	ORF40			---	−	379	ORF42
036	*p13*	+	280	+	277	hear40	+	278	ORF41	+	267	gp038	+	277	ORF43
037	*pif-2*	+	380	+	388	hear42	+	388	ORF43	+	378	gp039	+	388	ORF45
038	*ORF038*	+	1144	+	1279	hear44	+	1213	ORF45	+	848	gp041	−<br>+	81<br>220	ORF46,<br>ORF47 ‡
039	*ORF039*	−	227	−	257	hear45	−	260	ORF47	−	220	gp042	−	272	ORF50
040	*ORF040*	+	53			---			---			---			---
041	*odv-ec43*	+	414	+	353	hear48	+	353	ORF51	+	358	gp046	+	353	ORF53

Table S3. *Cont.*

SfGV VG008				Orthologous Genes											
				HearGV			PsunGV			SpliGV			XecnGV		
**ORF**	**Gene Name**	**Polar. (+/−)**	**Prot. Size (aa)**	**Polar. (+/−)**	**Prot. Size (aa)**	**NC_010240**	**Polar. (+/−)**	**Prot. Size (aa)**	**NC_013772**	**Polar. (+/−)**	**Prot. Size (aa)**	**NC_009503**	**Polar. (+/-)**	**Prot. Size (aa)**	**NC_002331**
042	*v-ubi*	−	82	−	77	hear47	−	77	ORF50	−	93	gp045	−	77	ORF52
043	*ORF043*	+	101	+	110	hear49	+	110	ORF52			---	+	110	ORF54
044	*39k*	−	291	−	293	hear50	−	298	ORF53	−	285	gp048	−	295	ORF55
045	*lef-11*	−	91	−	102	hear51	−	95	ORF54	−	98	gp049	−	102	ORF56
046	*ORF046*	+	317			---			---			---			---
047	*ORF047*	+	263	+	288	hear52	+	282	ORF55			---	+	278	ORF57
048	*dUTPase*	−	151			---			---	+	140	gp051			---
049	*ORF049*	+	287			---			---			---			---
050	*sod*	-	142	−	154	hear63	-	153	ORF64			---	−	153	ORF68
051	*DNA photolyase*	+	466			---			---	+	466	gp050			---
052	*bro-a*	−	252	+	463	hear133	−<br>+	496<br>241	ORF66,<br>ORF135	+	256	gp114	+	237	ORF130
053	*bro-b*	−	522	+<br>−	463<br>516	hear133,<br>hear54	−<br>+	496<br>241	ORF66,<br>ORF135	−<br>+<br>+	368<br>485<br>471	gp053,<br>gp115,<br>gp111	+<br>−	442<br>484	ORF131,<br>ORF60
054	*ORF054*	−	189			---			---			---			---
055	*ORF055*	+	184			---			---			---			---
056	*ORF056*	+	422	+	366	hear67	+	465	ORF72			---	+	458	ORF73
057	*bro-c*	−	717			---			---			---			---
058	*endonucl.*	+	93	+	97	hear69	+	95	ORF74			---	+	97	ORF75
059	*ORF059*	−	137			---			---			---			---

Table S3. *Cont.*

SfGV VG008				Orthologous Genes											
				HearGV			PsunGV			SpliGV			XecnGV		
**ORF**	**Gene Name**	**Polar. (+/−)**	**Prot. Size (aa)**	**Polar. (+/−)**	**Prot. Size (aa)**	**NC_010240**	**Polar. (+/−)**	**Prot. Size (aa)**	**NC_013772**	**Polar. (+/−)**	**Prot. Size (aa)**	**NC_009503**	**Polar. (+/-)**	**Prot. Size (aa)**	**NC_002331**
060	*bro-d*	+	267	+	273	hear71	+	270	ORF76			---	+	273	ORF76
061	*p74/pif-0*	+	704	+	710	hear72	+	710	ORF77	+	144	gp056§	+	710	ORF77
062	*ORF062*	−	93	−	104	hear73	−	111	ORF78			---			---
063	*p47*	+	394	+	394	hear74	+	394	ORF79	+	397	gp058	+	394	ORF78
064	*nudixPPi*	+	227	+	225	hear77	+	225	ORF82	+	222	gp059	+	225	ORF79
065	*p24*	+	176	+	182	hear78	+	171	ORF83	+	162	gp060	+	182	ORF80
066	*38.7k*	−	175	−	184	hear79	−	192	ORF84			---	−	184	ORF81
067	*lef-1*	−	238	−	238	hear80	−	238	ORF85	−	277	gp062	−	238	ORF82
068	*p10*	+	173	+	182	hear81	+	193	ORF86			---	+	182	ORF83
069	*pif-1*	+	541	+	540	hear82	+	541	ORF87	+	538	gp064	+	540	ORF84
070	*fgf-1*	−	228	−	232	hear83	−	233	ORF88	−	232	go066	−	232	ORF85
071	*chit-2a*	+	162	+	164	hear85	+	164	ORF90			---	+	164	ORF87
072	*chit-2b*	+	159	+	164	hear85	+	164	ORF90			---	+	164	ORF87
073	*lef-6*	−	99	−	99	hear86	−	99	ORF91			---	−	99	ORF88
074	*dbp*	−	281	−	277	hear87	−	277	ORF92	−	270	gp070	−	277	ORF89
075	*p48/p45*	+	372	+	372	hear90	+	372	ORF95	+	373	gp073	+	372	ORF91
076	*ORF076*	−	223	−	246	hear89	−	247	ORF94			---	-	246	ORF90
077	*p12*	+	111	+	121	hear91	+	121	ORF96	+	112	gp074	+	120	ORF92
078	*odv-ec42 /p40*	+	372	+	371	hear92	+	370	ORF97	+	369	gp075	+	372	ORF93
079	*p6.9*	+	58	+	58	hear93	+	60	ORF98			---	+	60	ORF94
080	*38k*	+	304	+	301	hear95	+	303	ORF100	+	299	gp077	+	301	ORF96

Table S3. *Cont.*

SfGV VG008				Orthologous Genes											
				HearGV			PsunGV			SpliGV			XecnGV		
**ORF**	**Gene Name**	**Polar. (+/−)**	**Prot. Size (aa)**	**Polar. (+/−)**	**Prot. Size (aa)**	**NC_010240**	**Polar. (+/−)**	**Prot. Size (aa)**	**NC_013772**	**Polar. (+/−)**	**Prot. Size (aa)**	**NC_009503**	**Polar. (+/-)**	**Prot. Size (aa)**	**NC_002331**
081	*lef-5*	−	260	−	268	hear94	−	266	ORF99	−	247	gp076	−	245	ORF95
082	*DNAhel-1*	+	1164	+	1160	hear97	+	1158	ORF102	+	1162	gp080	+	1159	ORF98
083	*pif-4*	−	157	−	157	hear96	−	157	ORF101	−	159	gp079	−	157	ORF97
084	*lef-7*	+	266	+	312	hear132			---			---	+	312	ORF129
085	*odv-e25*	−	219	−	220	hear98	−	219	ORF103	−	217	gp081	−	220	ORF99
086	*p33*	+	303	+	252	hear100	+	251	ORF105	+	251	gp083	+	251	ORF101
087	*p18*	−	158	−	158	hear99	−	158	ORF104	−	155	gp082	−	122	ORF100
088	*chaB*	−	79	−	87	hear103	−	87	ORF106			---	−	87	ORF102
089	*ORF089*	−	156	−	143	hear131	−	145	ORF132			---	−	143	ORF128
090	*bro-e*	−	537	+<br>−	463<br>516	hear133,<br>hear54	+	509	ORF137	+<br>+	471<br>485	gp111,<br>gp115	−<br>+	484<br>442	ORF60,<br>ORF131
091	*lef-4*	−	441	−	450	hear112	−	452	ORF114	−	438	gp086	−	447	ORF110
092	*vp39*	+	324	+	329	hear113	+	327	ORF115	+	317	gp087	+	329	ORF111
093	*odv-ec27 /odv-e27*	+	290	+	288	hear114	+	288	ORF116	+	284	gp088	+	288	ORF112
094	*bro-f*	−	370	−	372	hear116	−	404	ORF117	−	340	gp089	−	373	ORF113
095	*bro-g*	−	354	−	382	hear117	+	391	ORF118			---	+	397	ORF114
096	*ORF096*	+	385	+	386	hear118	−	423	ORF119			---	−	427	ORF115
097	*ORF097*	+	124	+	123	hear119	+	123	ORF120			---	+	123	ORF116
098	*ORF098*	−	198			---			---			---			---
099	*ORF099*	−	198			---			---			---			---
100	*tlp20*	+	179	+	159	hear122	+	164	ORF123	+	173	gp093	+	161	ORF119

Table S3. *Cont.*

SfGV VG008				Orthologous Genes											
				HearGV			PsunGV			SpliGV			XecnGV		
**ORF**	**Gene Name**	**Polar. (+/−)**	**Prot. Size (aa)**	**Polar. (+/−)**	**Prot. Size (aa)**	**NC_010240**	**Polar. (+/−)**	**Prot. Size (aa)**	**NC_013772**	**Polar. (+/−)**	**Prot. Size (aa)**	**NC_009503**	**Polar. (+/-)**	**Prot. Size (aa)**	**NC_002331**
101	*vp91/p95*	−	718	−	736	hear121	−	751	ORF122	−	557	gp092	−	741	ORF118
102	*ORF102*	+	191	+	187	hear123	+	187	ORF124	+	189	gp094	+	187	ORF120
103	*gp41*	+	294	+	290	hear124	+	290	ORF125	+	285	gp095	+	290	ORF121
104	*ORF104*	+	103	+	103	hear125	+	107	ORF126			---	+	103	ORF122
105	*vlf-1*	+	368	+	374	hear126	+	371	ORF127	+	372	gp097	+	373	ORF123
106	*ORF106*	+	85	+	85	hear128	+	85	ORF129	+	85	gp099	+	85	ORF125
107	*ORF107*	−	223	−	180	hear127	−	178	ORF128	−	181	gp098	−	180	ORF124
108	*ORF108*	+	145	+	145	hear129	+	145	ORF130	+	148	gp100	+	145	ORF126
109	*DNApol*	−	1065	−	1098	hear134	−	1094	ORF138	−	1035	gp101	−	1098	ORF132
110	*desmopl.*	+	637	+	661	hear135	+	663	ORF139	+	603	gp102	+	661	ORF133
111	*pif-6*	+	139	+	136	hear137	+	136	ORF141	+	129	gp104	+	120	ORF135
112	*lef-3*	−	350	−	351	hear136	−	345	ORF140	−	358	gp103	−	351	ORF134
113	*ORF113*	+	166	+	171	hear138	+	171	ORF142			---	+	171	ORF136
114	*iap-3*	+	289	+	284	hear139	+	290	ORF143	+	263	gp106	+	285	ORF137
115	*lef-9*	+	543	+	493	hear140	+	497	ORF145	+	503	gp107	+	493	ORF139
116	*fp*	+	170	+	147	hear141	+	70	ORF146	+	145	gp109	+	147	ORF140
117	*DNAligase*	−	534	−	527	hear142	−	552	ORF148	−	579	gp110	−	527	ORF141
118	*ORF118*	+	66			---			---			---			---
119	*ORF119*	+	66	+	66	hear144	+	66	ORF150			---	+	66	ORF143
120	*fgf-2*	−	387	−	409	hear145	−	404	ORF151	−	398	gp116	−	409	ORF144
121	*alk-exo*	+	405	+	457	hear146	+	473	ORF152	+	408	gp117	+	457	ORF145
122	*DNAhel-2*	+	470	+	455	hear147	+	457	ORF153	+	437	gp118	+	455	ORF146

Table S3. *Cont.*

SfGV VG008				Orthologous Genes											
				HearGV			PsunGV			SpliGV			XecnGV		
**ORF**	**Gene Name**	**Polar. (+/−)**	**Prot. Size (aa)**	**Polar. (+/−)**	**Prot. Size (aa)**	**NC_010240**	**Polar. (+/−)**	**Prot. Size (aa)**	**NC_013772**	**Polar. (+/−)**	**Prot. Size (aa)**	**NC_009503**	**Polar. (+/-)**	**Prot. Size (aa)**	**NC_002331**
123	*ORF123*	+	333	+	331	hear148	+	333	ORF154	+	326	gp120	+	309	ORF147
124	*lef-8*	−	859	−	859	hear149	−	860	ORF155	−	849	gp121	−	859	ORF148
125	*ORF125*	−	338			---			---			---			---
126	*odv-e66*	−	674	−	668	hear150	−	667	ORF156	+	715	gp125	−	668	ORF149
127	*enhancin-1*	−	867	−	865	hear152	+<br>−	901<br>828	ORF159,<br>ORF157			---	−	867	ORF152
128	*ORF128*	+	512	+	486	hear160	+<br>−	496<br>491	ORF164,<br>ORF021			---	+<br>−	486<br>492	ORF161,<br>ORF22
129	*ORF129*	+	334			---			---			---			---
130	*ORF130*	−	220	−	186	hear161	−	187	ORF165			---	−	186	ORF162
131	*ORF131*	+	161	+	118	hear163	+	118	ORF169			---	+	118	ORF165
132	*enhancin-2*	−	857	−	856	hear164	−	857	ORF170			---	−	856	ORF166
133	*ORF133*	+	149			---			---			---			---
134	*chit-2c*	+	162			---			---			---			---
135	*U-box /RING-like*	+	196	+	139	hear169	+	139	ORF173	+	165	gp127	+	139	ORF171
136	*ORF136*	−	417	−	378	hear170	−	373	ORF174	−	316	gp128	−	378	ORF172
137	*lef-10*	+	72	+	70	hear172	+	70	ORF176			---	+	70	ORF174
138	*ORF138*	−	67	−	67	hear171	−	67	ORF175			---	−	67	ORF173
139	*vp1054*	+	324	+	323	hear173	+	323	ORF177	+	320	gp130	+	323	ORF175
140	*ORF140*	+	61			---	+	60	ORF178			---			---

Table S3. *Cont.*

SfGV VG008				Orthologous Genes											
				HearGV			PsunGV			SpliGV			XecnGV		
ORF	Gene Name	Polar. (+/−)	Prot. Size (aa)	Polar. (+/−)	Prot. Size (aa)	NC_010240	Polar. (+/−)	Prot. Size (aa)	NC_013772	Polar. (+/−)	Prot. Size (aa)	NC_009503	Polar. (+/-)	Prot. Size (aa)	NC_002331
141	*ORF141*	+	136			---			---			---			---
142	*fgf-3*	+	301	+	351	hear176	+	307	ORF180	+	312	gp133	+	332	ORF178
143a	*ORF143a*	+	229			---			---			---			---
143b	*ORF143b*	+	206	+	197	hear177	+	210	ORF181			---	+	197	ORF179
144	*me53*	+	298	+	325	hear178	+	304	ORF182	+	289	gp134	+	325	ORF180
145	*ORF145*	+	99	+	109	hear179	+	107	ORF183			---	+	110	ORF181

‡ ORFs 47 and 48 of XcGV were concatenated. § ORF p74 in SpliGV is truncated.

Table S4. Homologous Regions in betabaculoviruses from Noctuide insects.

Virus	Homologous Region	Individual Repeats
SfGV VG008	hr1	19328 CAGAGATAACCTTCGATAAATTAATGTCGACCTTATCTTGGATTATTTT 19376
	hr2	39744 TATCAATATATCACATTATTTTAATGTCGATCCCATACCCAACTCTTTT 39792
		39843 ATCGTTAAAAACACATTGTTTTAATGTCGATCTTTGAGAGTGGGGTTTG 39795
		39880 ATCACAAAAAGCACATTGTTTTAATGTCGATATTTGGGATCCCCATTTT 39928
		39981 ATCACAAAAAGCACATTTTTTTAATGTCGCTCTAAAATATGTGTCTCCA 40029
		40025 CTCCAAAAACCTCAAAACACTTAATGTCGATCACATTATCTTTTTCTTA 40073
	hr3	46924 TCGTTTAACGTCACAATTTTTTAATGTCGCACTGGGACCATCTTTTTTT 46972
		47021 ATCGTAAAAAGCACAATTTTTTAATGTCGAGATTTTAGATCCCCATTTT 46973
		47055 ATCACAAAAAAGCACAATTTTTTAATGTCGAGATTCGACAACCCAAAATC 47103
		47229 ATCGCAAAAAGCACAATTTTTTAATGTCGCTCTTTGGAGGGTCCCCAAA 47181
		47279 TTCAAAACTTTTTACCCTCTTTAATGTCGGTATCAAAAAAAACACTGTT 47327

Table S4. *Cont.*

Virus	Homologous Region	Individual Repeats
SfGV VG008	hr4	53864 TTAAAACATTTTTACCCTGTTTAATGTCGGTATCAAAAAATACGGGTGT 53816
		53915 TCGTTGAAAAGCACCTTTTTTTAATGTCGATTTTGGGACAAAATGTTTT 53963
		54016 TTGTTAAAAAGCACATTTTTTTAATGTCGCTTTTTGGACTACCGTTTCA 54064
	hr5	87917 TTGAAAGTCGGTCGGGAGCGTTAATGTCGATCTTAAAAAGTGATATTTG 87869
		87912 TTTCAAAACCCCCAAAACTGTTAATGTCGATCTTGAATGTGTGAATAGG 87960
	hr6	122550 ATCACAAAAAGCACAATTTTTTAATGTCGATCTTCGACCAAACCATTTT 122598
		122648 AAAAGCACAATCACAATTTTTTAATGTCGATCCAACGGTTCCCGATGTT 122600
		122686 ATCACAAAAAGCACAATTTTTTAATGTCGATATTTTGGACCGGCTTTTA 122734
	hr7	128872 ATCGTTGACATCACTTTTTTTTAATGTCGATCCAACGGTTCCCGATGTT 128920
		128970 ATGTTAAAAAGCAACTTTTTTTAATGTCGCTCTTGGACGACACCGTTCG 128922
		129095 ATCATAAAAAGCACATTGTTTTAATGTCGATATTTTTTTTAAAAAAAGT 129047
		129128 ATCCTTAAAAACACATTGTTTTAATGTCGATCTTTGAGCATGCGCTCAA 129176
	hr8	133555 GACCATAAAGTCACATTGTTTTAATGTCGATATCTGAGACATGGTTTCA 133603
HearGV	hr1	36273 TTAATGTCGACCTTTTTGCACGTGTTTTAAAACACTGAAAAATTTAATGTCGA 36325
		36398 TTAATGTCGACCTTTTTGTAGATGTTTTAAAACTCTGAGAAATTTAATGTCGA 36450
		36522 TTAATGTCGAACCTTTTGAACCTTTTAAAAACTCTGAAAATTTTAATGTCGA 36573
		36643 TTAATGTTGATCTTTTTAGACCTTTCTAAAAACTCTGAACATATTAATATCGA 36695
		36765 TTAATGTCGACCGTTTTAGACCTTTTTAAAAACTCTGGAAATTTTAATATCGA 36817
	hr2	45107 TCGACATTAAAATTTCAGAGTTTTTAGAAAGGTCCAAAAAGATCGACATTAA 45159
		45228 TCGACATTAAAATTTCAGAGTTTTTAGAAAGGTCCAAAAAGATCGACATTAA 45280
		45349 TCGATATTAAAATTTCAGAGTTTTTAGAAAGGTCCAAAAAGATCGACATTAA 45401
		45472 TCGATATTAAATTTTTCGCGAGTTTTTGAGAGAACCAAAAAGGTCGACATTAA 45524
	hr3	59759 TCGATATTAAAATAAATAGAGTTTTAAAACTCTGGAAATTTTTAATGCTGA 59809
		59883 TCGATATTAAAATAAATAGAGTTTAAAAACTCTGGAAATTTTTGATGTTGA 59933
		60007 TCGACATTAAAATAAAAGCGCTTCAAAACTTTTGGAATTTTTTAATATCGA 60057
		60111 TCGACATTAAAATATTTCTATTTCAAAACTCTGTAAATTTTTAATATCGA 60160

Table S4. *Cont.*

Virus	Homologous Region	Individual Repeats
HearGV	hr4	65151 TTAATGTCGATCCCGTTCCCCGAAAGTTTTGAGACTGTTTTTTTAATGTCGA 65203
		65253 TTAATGTCGATCTTTTCCCCGGAAAGTTTTGAGACCCTATATTTTTAATGTCGA 65308
		65380 TTAATGTCGATCTTTTTCCCGAAAAGTTTTGGAGACTGTTTTTTTTAATGTCGA 65434
		65507 TTAATGTCGATTTGTAACGCTCAGAAGTTTTGGACTTGGTTTTGGTTAATGTCGA 65562
		65636 TTAATGTCGATCTTTTCGCCTCCCGGAGTTTTGAAGCTGTTTTTTTAATGTCGA 65689
		65763 TTAATGTCGACCTTTTTCCCAGAGTTTTAAAAACTTTTATTTTAATTTAATGTCGA 65818
		65867 TTAATATCGATATGTTGCTCTCGGAGTTTTAAACTTGGTTTTGGTTAATGTCGA 65920
	hr5	103745 TCGACATTAAAAAAAACAGGTTCTAAAACTTTGAGAATATTTTAATGTCGA 103795
		103881 TCGACATTAAAATATTTAGAGTTTAAAAACTCTTGAAATTTTTAATGTCGT 103931
	hr5a	108785 TTAATGTCGACCCCAAGTCCCAGAGTTTTTAAATCGAGCTCTTTGGGGTCGACATTAA 108842
	hr6	140761 TTAATGTCGACCCAAAATAGTGTTTTAAAAACTCTAAAATATTTTAATGTCGA 140813
		140885 TTAATGTCGACCTTTTTAGAGATGTTTTAAAACTCTGGAAATTTTAATGTCGA 140937
		141012 TTAATGTCGACCTTTTTAGAGATGTTTTAAAACTCTGGAAATTTTAATATCGA 141064
		141134 TTAATGTCGACCTTTTTGCAGATGTTTTAAAACTCTGAACAATTTAATATTGA 141186
	hr7	156952 TCGATATTAAAATATTTAAGGTTTTAAAACTCTGGAAATTTTTAATGTCGA 157002
		157081 TCGACATTAATTTTTTGGGAGTTTAAAACTCTGGAAATTTTTAATATCGA 157130
		157207 TCGACATTAAAAATTTGCAGAGTTTTAAAAGTCCCGAAAAATTAATATCGA 157257
	hr8	162512 TTAATGTCGATCTTTTTTCCCCGGAAAGTTTTTTGAGATTGTTTTTTTAATGTCGA 162567
		162638 GTAATGTCGATCTTTTTACCCAAAAAGTTTTTTAAACTCAATTTTTTTAATATCGA 162693
		162764 TTAATGTCGATCTTATTCTCCGGAAAGTTTTGAAGCTGTTTTTTTTAATGTCGA 162817
		162888 TTAATATCGACCTTTTCCCAGAGTTTTTAAAAGTTTCATTTTATTTTAATGTCGA 162942

Table S4. *Cont.*

Virus	Homologous Region	Individual Repeats
PsunGV	hr1	38849 TTAATATCGACCTTTTTCGAGTTGTTTAAAAACTTCCAAATATTAATGTCGA 38900<br>38972 TTAATATCGACCTTTTTCGAGACGTTTCAAAACTCTGAAAAAGTTAATATCGA 39024<br>39096 TTAATATCGACCTTTTTGGAGACGTTTCGAAACTCTGGAAATTTAATATCGA 39147<br>39218 TTAATATCGACCTTCTTTGAGAGGTTGTAAAAACTCTAGAAAATTAATATCGA 39270<br>39344 TTAATATCGATCTTTTTGGAGAGGTTGTAAAAACTCTGGAAACTTAATATCGA 39396
	hr2	49476 TCGACATTAAAATTTCCAGAGTTTTTGAAACGGTCTAAAAAGATCGATATTAA 48528<br>49601 TCGATATTAAAATTTTCAAGAGTTTTGAAACGTCCAAAAAAGATCGACATTAA 49653<br>49724 TCGACATTAAAGTTTTCAAGAGTTTAGAAACGGTCTAAAAAGATCGATATTAA 49776
	hr3	64357 TCGACATTAAAATAAAATATTTCAAAACTATTGAAAAATATTAATGTCGA 64406<br>64480 TTGACATTAAAATAAAAGTGTTTTAAAACTATTAGAAATTTTTAATATCAA 64530<br>64604 TTGATATTAAAAATTTATAATAGTTTTAAAACTATTGGAAATTTTTAATGTCGA 64657<br>64739 TCGACATTAAAATAAAAATGTTTCAAAACTATTGAAAATTTTAATATCAA 64779
	hr4	70499 TTAATATCGATTTTTTCCTCCCAGAAGTTTTGAAACTATTATTTTTGTTAATATCGA 70555<br>70659 TTAATATCGACCTTTTTTCAATAGTTTTACAAGTGTTTTTTATTAATGTCGA 70710<br>70784 TTAATGTCGATCTTTTTCCAGAAGTTTCAAGAGTGTTTTTTATTAATGTCGA 70835<br>70910 TTAATGTCGATCTTTTTCCAGAAGTTTCAAAAGTGTTTTTTATTAATGTAGT 70961<br>71034 TTAATATCGACCTTATACCGAGAGTTTTAAAACTACATTTTTATTAATGTCGA 71086
	hr5	108844 TCGATATTAAAATAAAACAGGTTTCAAAACTATTGGAAATTTTTAATATCGT 108895<br>108986 TCGATATTAACCAAAAATAATTATTTCAACACTCTAGGAATTTTTTAATATCGT 109039
	hr5a	113834 TTAATGTCGATCCCATCGCCCAGAGTTTTTAAATCGAGCTCTTTGGGATCGACATTAA 113891
	hr6	151064 TTAATGTCGACCTTTTTTAAAGGATTGAAAAAACTCCGCGTTATTGAAACAATATTAA 151121<br>151192 TTAATATCGACCCTATTTTTAAATTTCAAAAAACTCTGGAGGATAAAATCGATATTAA 151249<br>151316 TTAATATCGATCCCAATCCGGAGTTTTAAAACATTTCAAAAAGGTCGATATTAA 151369

Table S4. *Cont.*

Virus	Homologous Region	Individual Repeats
PsunGV	hr7	163831 TCGATATTAATCACCCATTCGGACTTTTTTGAAACTACAGAATAGGCTCGATATTAA 163887
		163959 TCGACATTAAATTTTTCGGAAGTTTTGAAACATGTGAAAAAAAGATCGACATTAA 164013
		164083 TCGATATTATAATTTCGGGAGTTTGAACATGTTAAAGAGATCGACATTAA 164132
		164341 TTAATGTCGATCTTTTTTAAACACTTTCAAAAGTCCGAGAATTTTAATGTCGA 164393
	hr8	169615 TTAATGTCGACCTTTTTCCTAAAGTTTTAAAATTGGATTTTGAATTAATATCGA 169668
		169741 TTAATGTCGATCTTTTCCCTATAGTTTTGAAATTAGTTTTTGCATTAATGTCGA 169794
		169868 TTAATGTCGATCTTTTTCCTATAGTTTTGAAATTGATTTCAAATTAATATCGA 169920
XecnGV	hr1	37403 TTAATGTCGACCTTTTAGTAGATGTTTTAAAACTCTGAAGAATTTAATGTCGA 37455
		37529 TTAATATCGACTTTTTAGAGGATGTTTTAAAACTCTGAAAAATTTAATGTCGA 37581
		37653 TTAATGTCGACCTTTTTAGAGATGTTTTAAAACTCCGGAAATTTTAATATCGA 37705
		37775 TTAATGTCGATCTTTTTAAACCTTTTTCAAAACTCTGAAAAATTTAATATCGA 37827
		37897 TTAATGTCGACCGTTTTGGACCTTTTTAAAAACTCTAAAAATTTTAATATCGA 37949
	hr2	46018 TCGACATTATAAATTTCTCAAAGTTTTTGAAACGCCTAAAAAATCGACATTAA 46070
		46146 TCGACATTAAAATTTCCAGAGTTTTAAAAAGGTATAAAAATATCGACATTAA 46197
		46267 TCGATATTAAATTTTTCAGAGTTTGAAAAGGTTTAAAACGGTCGACATTAA 46318
		46388 TCGATATTAAAATTTTCGCGAGTTTTTGAGAGACCCAAAAAGGTCGATATTAA 46440
		46515 TCGATATTAAAATTTTCGAGAGTTTTGAAACGGTCCAAAAAGGTCGACATTAA 46567
	hr3	63679 TCGATATTAAAATAAAATGAGTTTTAAAACTCTGGAAATTTTTAATGTTGA 63729
		63803 TCGATATTAAAACCAAATGAGTTTAAAAACTCTGGAAATTTTTAATGTCGA 63853
		63927 TCGACATTAAAAAAAAGCGCTTCAAAACTTTTGGGATTTTTTAATATCGA 63976
		64030 TCGATATTAAAAAATTTCTAGTTTAAAAACTCTGTAAATTTTTAATATCGA 64080

Table S4. *Cont.*

Virus	Homologous Region	Individual Repeats
XecnGV	hr4	69111 TTAATGTCGATCCCGTTTCCCCGAAAGTTTTGGGACTGTTTTTTTAATGTCGA 69164
		69235 TTAATGTCGATCTTTTCCCCGGAAAGTTTTGAGACTGTTTTTTTTAATGTCGA 69289
		69361 TTAATGTCGATTTGTAACGCTCAGAAGTTTTGGACTTGGTTTTGGTTAATGTCGA 69416
		69490 TTAATGTCGATCTTTTCGCCTCCCGGAGTTTTGAAGAGGTTTTTTTTAATGTCGA 69544
		69613 TTAATGTCGACCTTTTTTCCAGAGTTTTAAAAACTTTTATTTTGGTTTAATGTCGA 69668
		69732 TTAATATCGATATGTTGCTCTCGGAGTTTTAAAACTCTAAATTATTTTAATGTCGA 69787
	hr5	106427 TCGACATTAAAAAAAACAGGTTCTAAAACTTTGAGAATATTTTAATGTCGA 106477
		106552 TCGATATTAAAATATTTAGAGTTTTAAAACTCTGGAAATTTTTAATGTCGT 106602
		106698 TCGATATTAAATTAGAAAAAACTATTAAAAACTCTGGTGATTTTAATGTCGA 106749
	hr5a	111546 TTAATGTCGACCCCAAGTCCCAGAGTTTTAAATCGAGCTCTTTGGGGTCGACATTAA 111603
	hr6	146419 TTAATGTCGACCCAAAATAGAGTTTTAAAAAATCTGAAATATTTTAATGTTGA 146471
		146543 TTAATGTCGACCTTTTAGAGATGTTTTAAAACTCTGGAAATTTTAATGTCGA 146594
		146669 TTAATGTCGACCTTTTTAGAGATGTTTTAAAACTCTGAAAATTTTAATATCGA 146721
		146791 TTAATGTCGACCTTTTTGCAGATGTTTTGAAACTCTGAAAAATTTAATGTCGA 146843
	hr7	162098 TCGATATTAAAATATTTAGAGTTTTAAAACTCTGGGAATTTTTAATGTCGA 162148
		162227 TCGATATTAAAAATTTCCAGAGTTTTAAAACTCCCAAAAAATTAATGTTGA 162277
		162352 TCGACATTAATTTTTGGAAGTTTTAAAACTCTGGAAATTTTTAATATCGA 162401
		162455 TCGACATTAATTTTTTGAGAGTTTTAAAACTCTGGAAATTTTTAATGTCGA 162505
		162581 TCGACATTAAAATTTTCCAGAGTTTTAAAACTCCCGAAAAATTAATATCGA 162631
	hr8	171502 TTAATGTCGATCTTTTTCCCCGGAAAGTTTTGAGACCTTATATATTTAATGTCGA 171556
		171627 TTAATGTCGATCTTTTTACCCAAAAGTTTTAAAACTCAATTTTTTTAATGTCGA 171681
		171752 TTAATGTCGATCTTTTCGCCTCCCGGAGTTTTGAAGCTGTTTTTTTTAATGTCGA 171806
		171877 TTAATATCGACCTTTTCCCAGAGTTTATAAAAGTTTCATTTTATTTTAATGTCGA 171931

Underlined the consensus nona- or decanucleotide.
